# YY1 lactylation in microglia promotes angiogenesis through transcription activation-mediated upregulation of FGF2

**DOI:** 10.1186/s13059-023-02931-y

**Published:** 2023-04-21

**Authors:** Xiaotang Wang, Wei Fan, Na Li, Yan Ma, Mudi Yao, Guoqing Wang, Siyuan He, Wanqian Li, Jun Tan, Qi Lu, Shengping Hou

**Affiliations:** 1 The First Affiliated Hospital of Chongqing Medical University, Chongqing, China; 2 Chongqing Key Laboratory of Ophthalmology, Chongqing, China; 3 Chongqing Eye Institute, Chongqing, China; 4 Chongqing Branch of National Clinical Research Center for Ocular Diseases, Chongqing, China; 5 School of Basic Medical Sciences, Chongqing Medical University, Chongqing, 400016 China; 6 The Affiliated Eye Hospital, Nanjing Medical University, Nanjing, China; 7 The Children’s Hospital of Chongqing Medical University, Chongqing, China; 8 Beijing Institute of Ophthalmology, Beijing Tongren Eye Center, Beijing Tongren Hospital, Capital Medical University, Beijing Ophthalmology & Visual Sciences Key Laboratory, Beijing, 100730 China

**Keywords:** Angiogenesis, Retinal microglia, Posttranslational modifications (PTMs), Lactylation, YY1

## Abstract

**Background:**

Ocular neovascularization is a leading cause of blindness. Retinal microglia have been implicated in hypoxia-induced angiogenesis and vasculopathy, but the underlying mechanisms are not entirely clear. Lactylation is a novel lactate-derived posttranslational modification that plays key roles in multiple cellular processes. Since hypoxia in ischemic retinopathy is a precipitating factor for retinal neovascularization, lactylation is very likely to be involved in this process. The present study aimed to explore the role of lactylation in retinal neovascularization and identify new therapeutic targets for retinal neovascular diseases.

**Results:**

Microglial depletion by the colony-stimulating factor 1 receptor (CSF1R) inhibitor PLX3397 suppresses retinal neovascularization in oxygen-induced retinopathy. Hypoxia increased lactylation in microglia and accelerates FGF2 expression, promoting retinal neovascularization. We identify 77 sites of 67 proteins with increased lactylation in the context of increased lactate under hypoxia. Our results show that the nonhistone protein Yin Yang-1 (YY1), a transcription factor, is lactylated at lysine 183 (K183), which is regulated by p300. Hyperlactylated YY1 directly enhances FGF2 transcription and promotes angiogenesis. YY1 mutation at K183 eliminates these effects. Overexpression of p300 increases YY1 lactylation and enhances angiogenesis in vitro and administration of the p300 inhibitor A485 greatly suppresses vascularization in vivo and in vitro.

**Conclusions:**

Our results suggest that YY1 lactylation in microglia plays an important role in retinal neovascularization by upregulating FGF2 expression. Targeting the lactate/p300/YY1 lactylation/FGF2 axis may provide new therapeutic targets for proliferative retinopathies.

**Supplementary Information:**

The online version contains supplementary material available at 10.1186/s13059-023-02931-y

## Background

Retinopathy of prematurity (ROP) is a major cause of infantile visual impairments and blindness [1, 2]. High oxygen therapy for preterm infants leads to the interruption of normal vessel development and even the loss of well-developed vessels in the retina since preterm infants have incompletely vascularized retinas. After returning to a typical room air environment, which is relatively hypoxic, the incomplete retinal vascular network cannot meet the increasing metabolic demand. The release of multiple angiogenic factors increases in the hypoxic retina and stimulates pathological vessel formation at the junction between the vascular and avascular areas [1–3]. In addition, the newly formed vessels are involved in complex retinal pathological changes, including vascular leakage, inflammation, and blood-retina barrier (BRB) damage, causing the formation of hemorrhages and detachment of the retina, ultimately leading to visual impairments and even permanent blindness [2, 4]. Anti-VEGF agents are widely used in clinical practice, but their efficacy is sometimes limited [5, 6]. Repeated intraocular injections may lead to various adverse effects [7]. Laser photocoagulation is applied in some cases, but it is a destructive method that carries significant risk [6, 8]. It is important to further elucidate the mechanisms of retinal neovascularization and discover new therapeutic targets.

Microglia, the resident immune cells in the central nervous system (CNS) and retina, have been reported to play crucial roles in angiogenesis and vasculopathy [9–11]. Microglia maintain bidirectional communication with endothelial cells (ECs) and are the first immune cells to be activated during hypoxia [12]. Retinal hypoxia leads to pathological angiogenesis and microglia are attracted to the site, localize closely with newly formed vessels, and act as important regulators [10, 13]. Activated microglia actively participate in the development of OIR, and exert different functions at different stages [10]. Previous evidence indicates the pivotal proangiogenic role of microglia, but the molecular mechanism remains obscure.

Lactate, a compound produced during the Warburg effect, was previously defined as an energy source and metabolic byproduct that can be generated under hypoxic conditions. Recently, some new functions of lactate have been continuously discovered [14–17]. Lactylation is a novel lactate-derived posttranslational modification (PTM) of histone proteins originally identified by Zhang et al. [18]. It functions as a vital epigenetic regulator in many cellular processes. For example, evidence has shown that lactylation can regulate macrophage polarization during bacterial infection, accelerate ocular melanoma tumorigenesis, and facilitate cellular reprogramming [18–21]. However, the role of lactylation in angiogenesis has not been clarified. Since most retinal vasculopathies are induced by hypoxia, including OIR, lactylation may be involved in retinal neovascularization and exploring the mechanism is interesting.

In the current study, we demonstrate that hypoxia-induced hyperlactylation in microglia plays pivotal roles in retinal neovascularization. We performed lactylome and proteomic analyses and found 67 hyperlactylated proteins in microglia under hypoxia. Further mechanistic studies showed that the lactylation of nonhistone YY1 is increased in response to hypoxia and is regulated by p300. YY1 directly binds to the promoter of FGF2 and promotes FGF2 transcription with hyperlactylation and then contributes to neovascularization. Inhibiting the Kla writer p300 reduces the lactylation of YY1 and suppresses angiogenesis in vivo and in vitro. Collectively, we evaluated the mechanistic linkage between hypoxia, lactylation, and microglia-mediated neovascularization and provide potential therapeutic targets for retinal neovascular diseases.

## Results

### Retinal microglia are essential for retinal neovascularization

**Fig. 1:**
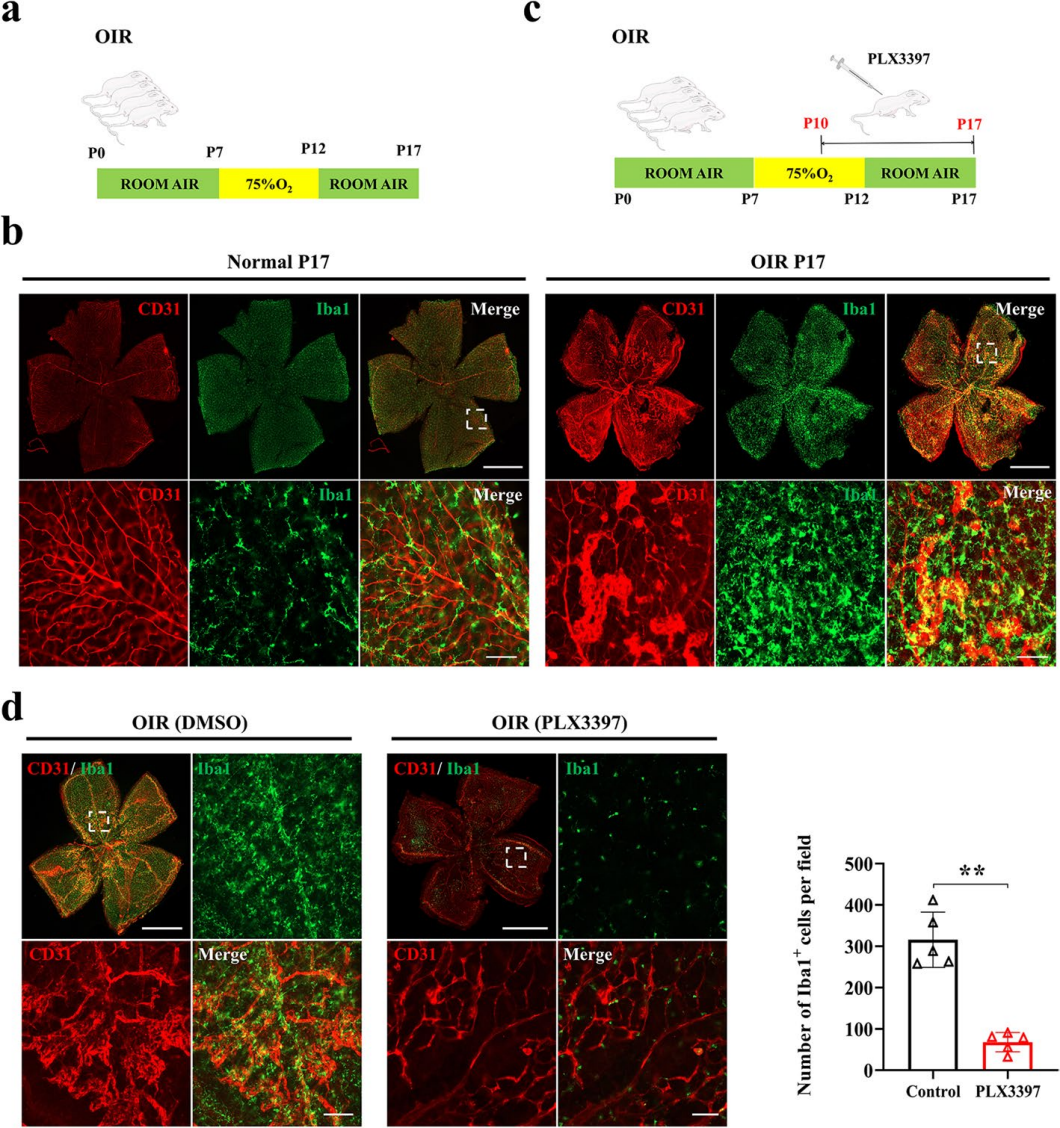
Retinal microglia are essential for retinal neovascularization. **a** OIR modeling flow chart. **b** Iba-1 (microglial marker) and CD31 (endothelial cell marker) double-stained confocal images of retinas in normoxia control (Normal P17) and OIR mice (OIR P17). **c** PLX3397 dosing flow chart. **d** Iba-1 and CD31 double-stained retinal flat mounts (OIR (DMSO) and OIR (PLX3397)). The number of Iba1 positive cells was recorded and analyzed by CellSens Dimension software (*n* = 5 mice per group). Scale bars, 1000 μm (upper panel of b and top left panel of d) or 50 μm (lower panel of **b** and bottom right panel of **d**). **p < 0.01

To evaluate the role of microglia in the vascular pathogenesis of retinopathy, a widely used OIR mouse model that recapitulates human proliferative retinopathies such as ROP was developed as shown in the flow chart (Fig. 1a). After modeling, retinal microglia proliferated substantially and gathered around the site of neovascularization in the OIR retina (Fig. 1b). To assess whether microglial depletion could affect OIR progression, we utilized a CSF1R inhibitor (PLX3397), which was shown to induce significant microglial ablation [22, 23]. The dosing flow chart is shown in Fig. 1c (injection from P10 until P17). After administration, we observed that pathological angiogenesis was greatly suppressed along with the depletion of microglia (Fig. 1d). These results demonstrate the important role of microglia in retinal neovascularization.

### Elevated lactate and lactylation levels are associated with retinal neovascularization

**Fig. 2:**
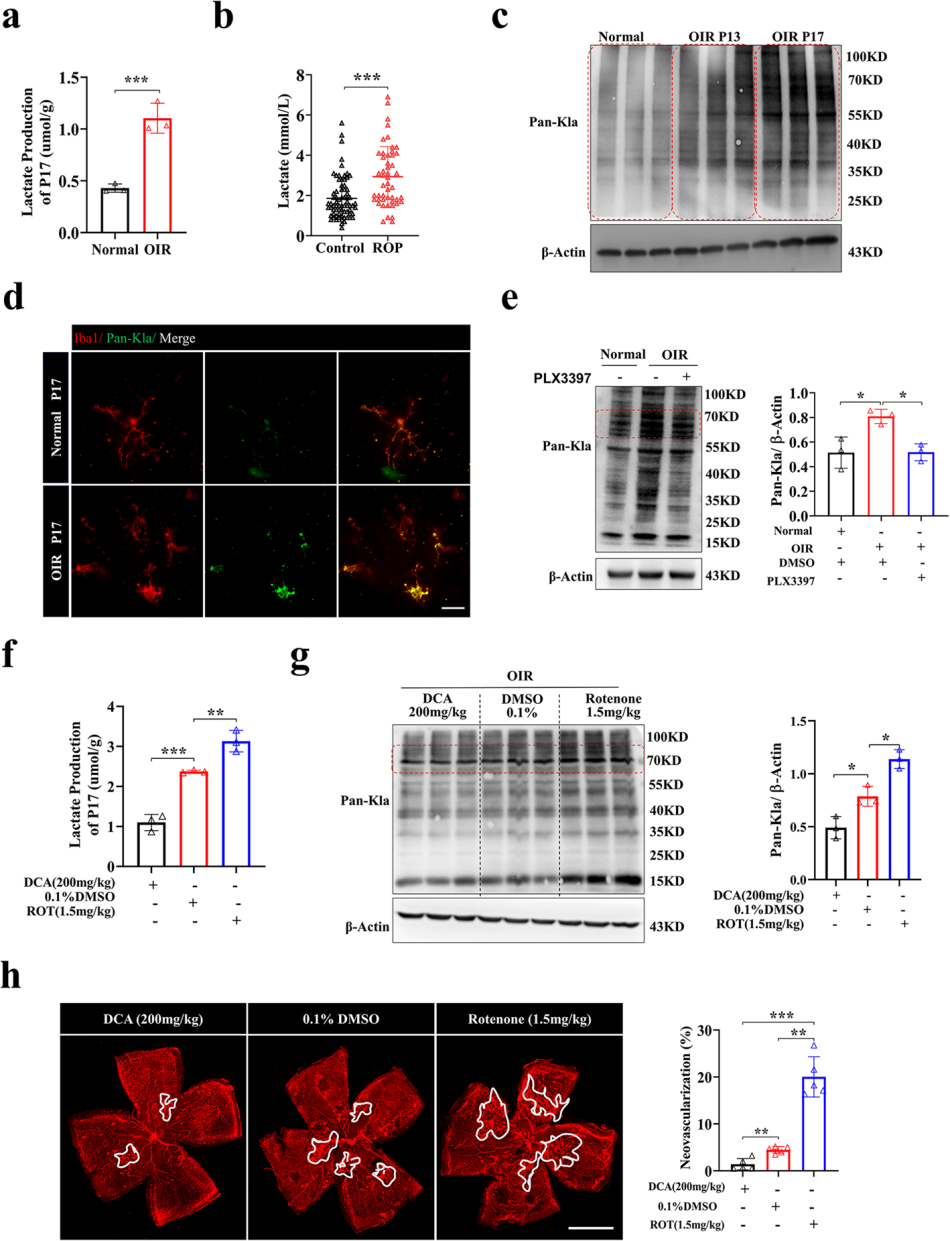
Elevated lactate and lactylation levels are associated with retinal neovascularization. **a** Retinal lactate quantification in normoxia control mice (Normal) and OIR mice (OIR) at P17 (*n* = 3 independent experiments, at least 6 mice each group). **b** Blood lactate quantification for ROP (ROP) (*n* = 49) and non-ROP (Control) (*n* = 72) infants (The normal range is between 0.7 ~ 2.1 mmol/L). **c** Pan-Kla in the retinas of normal control (Normal), OIR (OIR P13), and OIR (OIR P17) mice (*n* = 3 per group). **d** Iba-1 and Pan-Kla double-stained retinal flat mounts of normoxia control (Normal P17) and OIR mice (OIR P17); scale bar, 20 μm. **e** Pan-Kla in the retinas of normal control (Normal), OIR (DMSO) and OIR (PLX3397) mice (*n* = 3 per group). **f** Lactate production of retinal samples in OIR mice (OIR P17) treated with DCA (200 mg/kg), DMSO (0.1%), and rotenone (1.5 mg/kg) (*n* = 3 independent experiments, at least 6 mice each group). **g** Quantification of Pan-Kla in the retinas of OIR mice (OIR P17) treated with DCA (200 mg/kg), DMSO (0.1%), and rotenone (1.5 mg/kg) were analyzed by Western blotting (*n* = 3 per group). **h** Confocal images of CD31-stained retinal flat mounts in OIR P17 mice treated with DCA (200 mg/kg), DMSO (0.1%), and rotenone (1.5 mg/kg). The relative proportion of neovascularization in the retina was calculated and measured by ImageJ; scale bar, 1000 μm (*n* = 5 mice per group). *p < 0.05; **p < 0.01; ***p < 0.001

Lactylation is derived from lactate and participates in multiple cellular processes [18, 19]. First, to confirm whether lactate and lactylation are involved in proliferative retinopathies induced by hypoxia, we examined the lactate content and pan lysine lactylation (Pan-Kla) levels in OIR mice. The lactate levels were upregulated in the retina of OIR mouse on P17 (the peak of neovascularization in OIR) compared with the age-matched control group (Fig. 2a), in line with the clinical data that the lactate content in the blood of ROP infants was significantly increased compared with that in non-ROP infants (Fig. 2b). Meanwhile, Pan-Kla levels were upregulated in the OIR group compared with the control group (Fig. 2c). We then wondered whether the lactylation of microglia plays an important role in retinal vascular disease. Lactylation was upregulated in retinal microglia of the OIR mice (Fig. 2d). Notably, the level of Pan-Kla in the retina was relatively downregulated with the depletion of microglia (Fig. 2e).

To examine whether lactylation contributes to the vascular pathogenesis of retinopathy, we applied two compounds for in vivo verification experiments, sodium dichloroacetate (DCA) and rotenone, which have been shown to regulate lactylation by regulating lactate production [18]. DCA can reduce the production of lactate by inhibiting the activity of pyruvate dehydrogenase kinase, and rotenone is an inhibitor of the mitochondrial respiratory chain complex that causes cells to tend to undergo glycolysis and increases the content of lactate [24, 25]. After compound treatment, we observed that lactate and lactylation levels were decreased in the DCA group and increased in the rotenone group (Fig. 2f, g), which was accompanied with alleviated retinal neovascularization in the DCA group and aggravated neovascularization in the rotenone group (Fig. 2h). These results indicate the important role of lactylation in retinal neovascularization.

### Hyperlactylation of microglia promotes angiogenesis in vitro

**Fig. 3:**
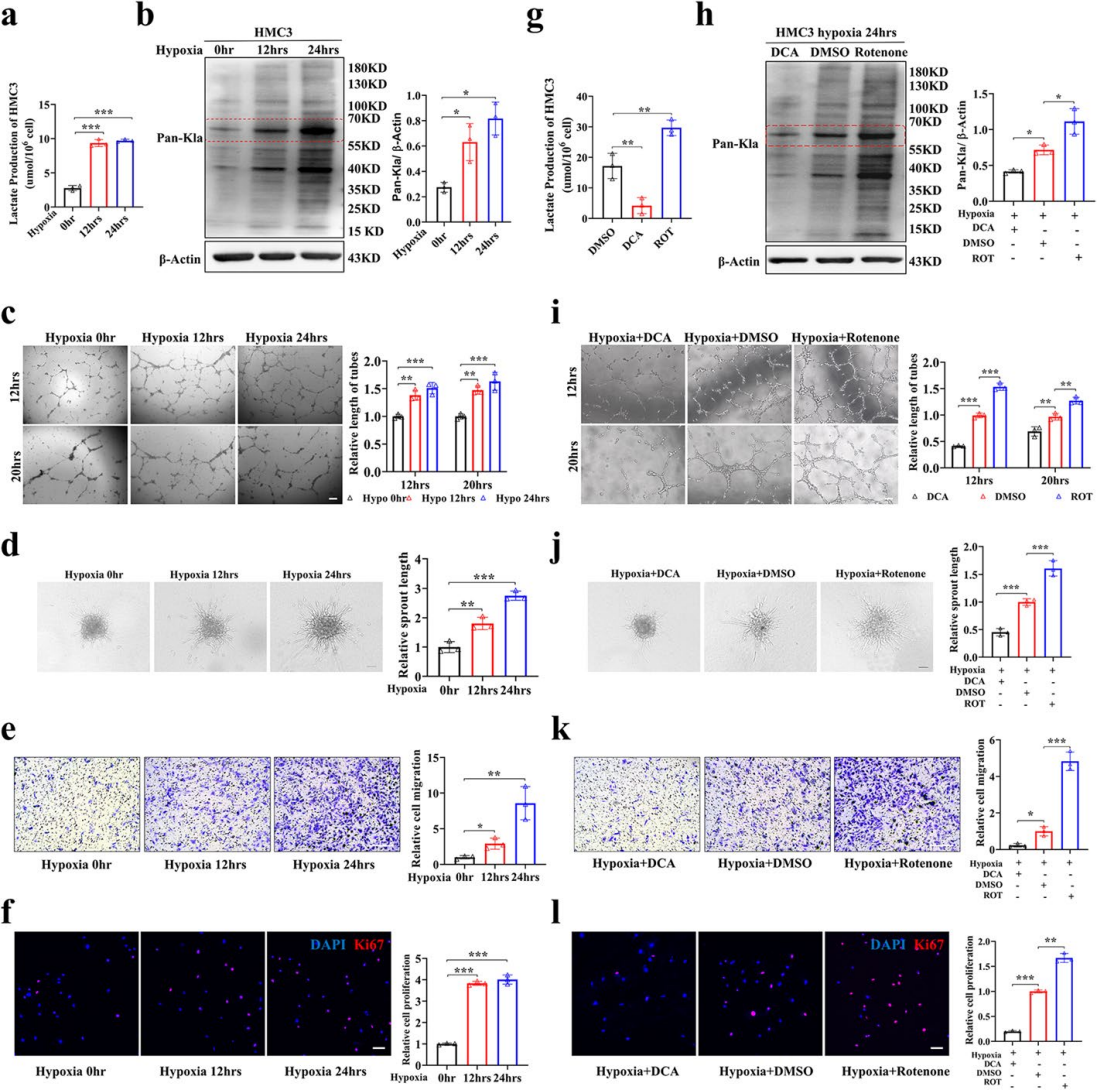
Hyperlactylation of microglia promotes angiogenesis in vitro. **a** Lactate production of HMC3 cells in the three groups treated with hypoxia for 0, 12, and 24 h (*n* = 3 independent experiments, at least 6 mice each group). **b** Quantification of Pan-Kla in the HMC3 cells of hypoxia 0 h, hypoxia 12 h, and hypoxia 24 h were analyzed by Western blotting (*n* = 3 per group). **c** Tube formation analysis. HMC3 cells were pretreated with 1% O_2_ or normoxia for 24 h and then cocultured with HRMECs; tube formation was assayed 12 h and 20 h after cell seeding (*n* = 3 independent experiments, 3 images for each group); scale bar, 50 µm. **d** HRMECs angiogenic capacity was evaluated by spheroid-sprouting assay. Representative images of spheroid sprouting were analyzed (*n* = 3 independent experiments, 3 images for each group); scale bar, 50 µm. **e** HRMECs migration was evaluated by Transwell assay (*n* = 3 independent experiments, 3 images for each group); scale bar, 25 µm. **f** HRMEC proliferation was evaluated by Ki67 staining (*n* = 3 independent experiments, 3 images for each group); scale bar, 100 µm. **g**, **h** Quantification of lactate and Pan-Kla in HMC3 cells in the three groups treated with DCA (20 mM), DMSO, and rotenone (50 nM) under hypoxia for 24 h (*n* = 3 per group). **i–l** HMC3 cells were pretreated with 1% O_2_ for 24 h with DCA, DMSO, or rotenone. Then HRMECs were cocultured with the pretreated microglia. The tube formation, spheroid sprouting, migration, and proliferation assays were performed as shown in the “Methods” (*n* = 3 independent experiments, 3 images for each group); scale bars, 100 μm (**k**) or 50 μm (**i**, **j**, and **l**). *p < 0.05; **p < 0.01; ***p < 0.001

Since hypoxia is important in the process of angiogenesis, we exposed human microglial clone 3 (HMC3) cells to hypoxia to explore the role of lactylation in microglia. As the degree of hypoxia increased, the lactate content of HMC3 microglia increased, and the level of lactylation was also elevated, especially for proteins in the range of 55–70 kD (Fig. 3a, b). The proliferation and migration of endothelial cells are vital for the formation of vascular networks. We wondered whether microglia with increased lactylation could influence the angiogenic abilities of endothelial cells. We cocultured HMC3 microglia from normoxic or hypoxic groups with human retinal microvascular endothelial cells (HRMECs) (Additional file 1: Fig. S1a). As the hypoxic time of HMC3 cells increased, the capabilities of tube formation, spheroid sprouting, migration, and proliferation of HRMECs were enhanced (Fig. 3c–f). To further confirm the significant role of the lactate/lactylation levels of microglia in the pathogenesis of angiogenesis, HMC3 microglia were treated with DCA and rotenone and then the levels of lactate/lactylation were measured. The lactate/lactylation levels of HMC3 microglia were decreased in the DCA group and increased in the rotenone group (Fig. 3g, h). We then cocultured HMC3 microglia from the DCA/DMSO/rotenone groups with HRMECs to explore the influence on HRMECs. The tube formation, spheroid sprouting, migration, and proliferation abilities of HRMECs cocultured with HMC3 cells in the DCA group were weakened, while they were enhanced in the rotenone group (Fig. 3i–l). These results show that elevated lactylation in microglia is responsible for promoting the angiogenic abilities of endothelial cells.

### The lactylation of YY1 in microglia plays an important role in regulating angiogenesis

**Fig. 4:**
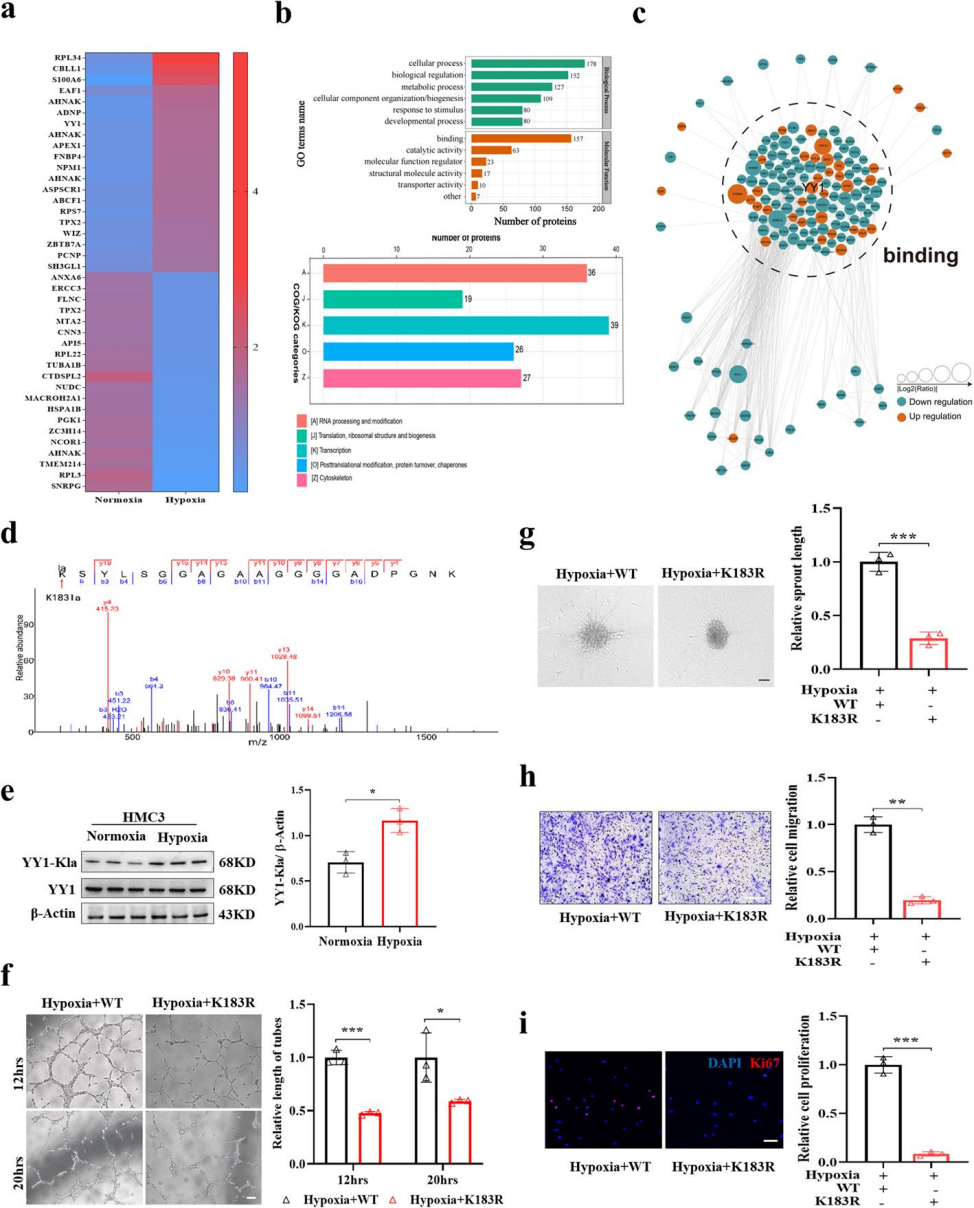
The lactylation of YY1 in microglia plays an important role in regulating angiogenesis. **a** Heatmap shows the top 20 DELPs with increased and decreased lactylation under hypoxia for 24 h. **b** The enrichment analysis of DELPs. **c** Using the STRING database, DELPs specifically target the binding process. The node size corresponds to the relative fold-change of lactylation. **d** Collision-induced dissociation (CID) analysis was used to determine the modification sites. The MS/MS spectrum of modified “(Kla)SYLSGGAGAAGGGGADPGNK” is shown. **e** Hypoxia increases YY1 lactylation. Lactylation of YY1 in HMC3 cells under normoxia or hypoxia for 24 h was detected by YY1-Kla antibody (*n* = 3 per group). **f–i** HMC3 cells overexpressing WT or K183R YY1 were pretreated with 1% O_2_ for 24 h. Then HRMECs were cocultured with the pretreated HMC3 cells. The tube formation, spheroid sprouting, migration, and proliferation assays were performed as shown in the “Methods” (*n* = 3 independent experiments, 3 images for each group); scale bars, 50 μm (**f**, **g**, and **i**) or 100 μm (**h**). *p < 0.05; **p < 0.01; ***p < 0.001

To characterize the landscape of lactylation in microglia under hypoxia, we treated HMC3 cells with normoxia or hypoxia for 24 h and then applied lactylome analysis by the 4D label-free platform method to identify the differentially lactylated proteins. For the details of the lactylome data, a total of 6021 peptides and 3071 lactylated peptides were identified. In total, 3093 lactylated sites in 751 proteins were identified in HMC3 cells, with a localization probability > 0.75 and a 1% FDR (Additional file 1: Fig. S2a). Quality control revealed that the distribution of peptides was in a reasonable range (Additional file 1: Fig. S2b). We identified 77 sites of 67 proteins with increased lactylation and 190 sites of 162 proteins with decreased lactylation under hypoxic conditions as differentially expressed lactylated proteins (DELPs) (Additional file 1: Fig. S2c). The relative quantification of the top 20 proteins with increased or decreased lactylation is shown in Fig. 4a. Cluster analysis of the identified DELPs was performed to characterize the function of these lactylated proteins. The classification analysis indicated that most of the DELPs exert functions in the nucleus (Additional file 1: Fig. S2d) and have a potential role in regulating DNA transcription (Fig. 4b). Using the Search Tool for the Retrieval of Interacting Genes/Proteins (STRING) database, the protein interaction networks of DELPs suggested that DELPs mainly function in the binding process, which corresponds with enriched pathways (Fig. 4c).

Among these DELPs, YY1 immediately caught our attention since its lactylation level was largely upregulated (fold-change 3.56) and YY1 is a multifunctional transcription factor that has a certain regulatory effect on angiogenesis [26]. In addition, the observed band size of YY1 is 68 kD, which is consistent with the range 55–70 kD that is mainly regulated by hypoxia or treatment with DCA and rotenone according to our previous results (Figs. 2c, g and 3b, h). We observed colocalization of YY1 and Pan-Kla with double-labeled immunofluorescence in hypoxic HMC3 microglia (Additional file 1: Fig. S2e). The proteomic analysis identified only one lactylated lysine residue in YY1 (K183), showing a characteristic tandem mass spectrometry (MS/MS) spectrum including C-terminal y-ions and amino-terminal b-ions (Fig. 4d). To verify whether YY1 lactylation was regulated under hypoxia, we used specialized YY1-K183la antibody to detect the level of YY1 lactylation. Consistent with the sequencing results, YY1 lactylation was largely increased under hypoxia, and we observed no difference in YY1 expression levels (Fig. 4e). These results were verified by IB after IP assays (Additional file 1: Fig. S2f).

We next mutated lysine (K)183 of YY1 to arginine (R), which mimics the delactylated state of the protein, by transfecting HMC3 microglia with lentivirus containing cDNA of the Flag-tagged YY1 WT or YY1 K183R mutant. Flag-YY1 was overexpressed in both the WT and K183R mutant groups (Additional file 1: Fig. S2g-h). YY1 lactylation levels were decreased in K183R mutant hypoxia treated HMC3 microglia (Additional file 1: Fig. S2h). Compared with the WT group, HRMECs cocultured with K183R mutant HMC3 microglia showed weakened tube formation, spheroid sprouting, migration, and proliferation capabilities (Fig. 4f–i). Taken together, these data indicate that lactylation of YY1 in microglia functions as a regulator of angiogenesis.

### YY1 lactylation contributes to angiogenesis by regulating FGF2 expression

**Fig. 5:**
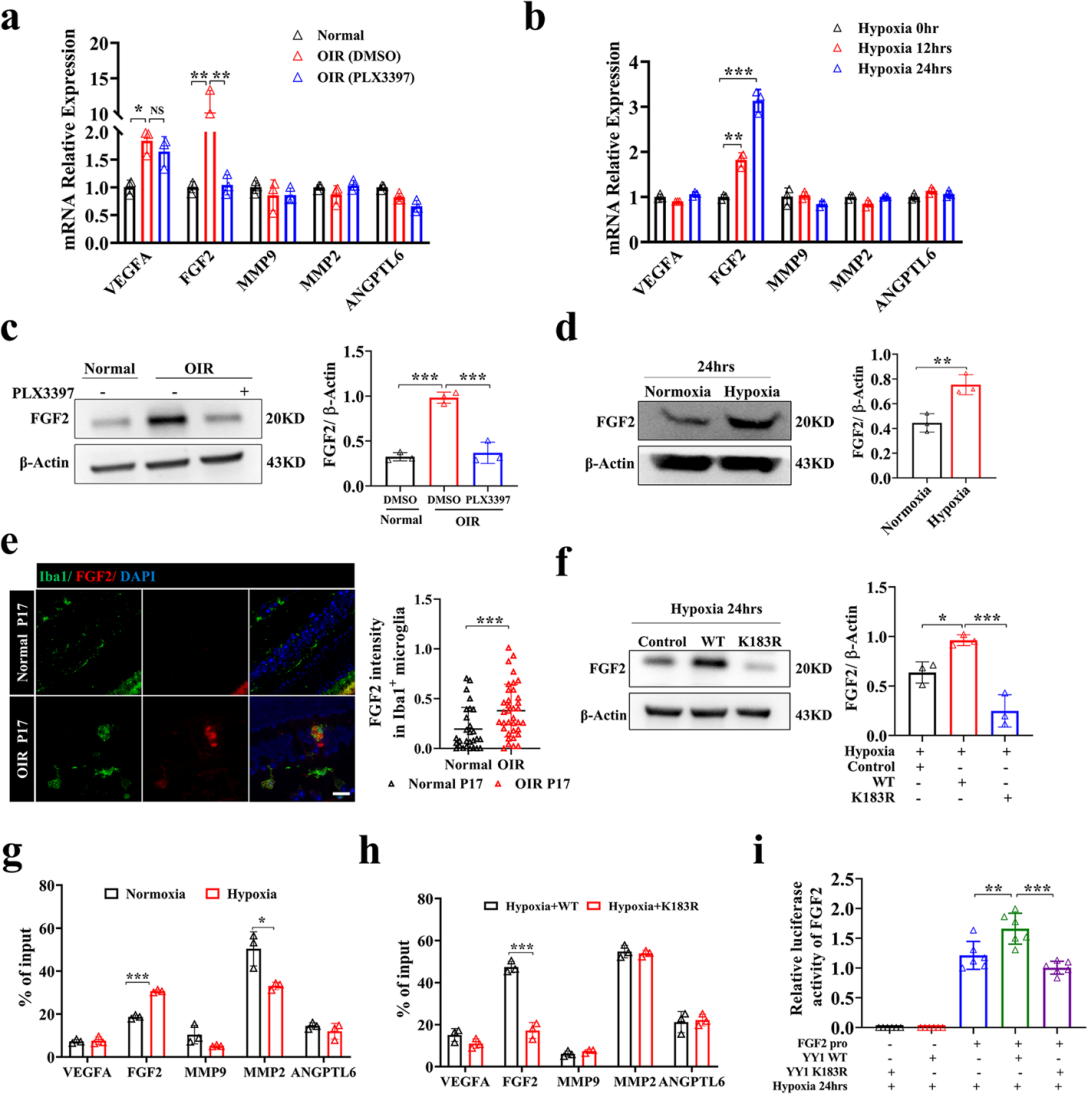
YY1 lactylation contributes to angiogenesis by regulating FGF2 expression. **a** The mRNA expression of VEGFA, FGF2, MMP2, MMP9, and ANGPTL6 in the retinal tissue of normal control (Normal), OIR (DMSO), and OIR (PLX3397) mice (*n* = 3 per group). **b** The mRNA expression of VEGFA, FGF2, MMP2, MMP9, and ANGPTL6 in HMC3 cells in the three groups subjected to hypoxia for 0, 12, and 24 h (*n* = 3 per group). **c** The protein expression of FGF2 in the retinas of normal control (Normal), OIR (DMSO), and OIR (PLX3397) mice (*n* = 3 per group). **d** The protein expression of FGF2 in the HMC3 cells subjected to hypoxia for 0 and 24 h (*n* = 3 per group). **e** Representative images of FGF2 co-stained with microglia (Iba1) in retina of OIR mice (OIR P17) and control mice (Normal P17), with quantification of FGF2 intensity (*n* = the number of Iba1^+^ cells); scale bar, 20 μm. **f** The protein expression of FGF2 in HMC3 cells exposed to hypoxia 24 h (Hypoxia Control), hypoxia for 24 h + YY1 WT transfection (Hypoxia + WT), and hypoxia for 24 h + YY1 K183R transfection (Hypoxia + K183R) (*n* = 3 per group). **g** ChIP-qPCR analysis of the indicated promoters was performed by using of YY1 antibody in HMC3 cells treated with normoxia and hypoxia (*n* = 3 per group). **h** ChIP-qPCR analysis of the indicated promoters was performed by using of YY1 antibody in HMC3 cells overexpressing WT and K183R YY1 (*n* = 3 per group). **i** The luciferase activity of the FGF2 promoter driven reporter vector was measured in response to YY1 WT or K183R cotransfection under hypoxia (*n* = 6 per group). NS, non-significance; *p <0.05; **p < 0.01; ***p < 0.001

To identify the potential angiogenic factors that are involved in microglia-mediated angiogenesis, we reverse-transcribed mRNA isolated from normoxic, hypoxic, and microglia-depleted retinas in vivo and HMC3 microglia in vitro and compared the expression levels of some classic angiogenic-related genes (VEGFA/FGF2/MMP9/MMP2/ANGPTL6) [27–32]. The FGF2 and VEGFA mRNA expression levels were significantly elevated in OIR mice compared with the control group, while only FGF2 levels decreased notably at both the mRNA and protein levels after microglial depletion (Fig. 5a, c). Similarly, hypoxia increased FGF2 mRNA expression within 12 h of hypoxia exposure, and the levels remained elevated until 24 h in HMC3 microglia (Fig. 5b, d). FGF2 expression was upregulated in OIR retinal microglia (Fig. 5e). Consistent with previous studies, these results indicate that FGF2 is important in microglia-mediated neovascularization [9]. DCA and rotenone were used in vivo and in vitro to explore whether lactate/lactylation affects angiogenesis by regulating the expression of angiogenic factors. In the OIR model treated with DCA and rotenone, the expression of FGF2 was decreased in the DCA group but increased in the rotenone group at P17 (Additional file 1: Fig. S3a). Similar results were found in HMC3 microglia exposed to hypoxia with DCA or rotenone (Additional file 1: Fig. S3b).

YY1 contains four C2H2 zinc fingers that are used to bind a specific DNA sequence located in many promoters and enhancers, promoting or repressing transcription [33, 34]. We detected lower expression of FGF2 at both the mRNA and protein levels in the YY1 K183R mutant group, while there was no difference in other angiogenic factors (Fig. 5f and Additional file 1: Fig. S3c). To explore the mechanism between YY1 lactylation and FGF2 expression, we searched the Cistrome Data Browser database and found that YY1 may directly promote the transcription of FGF2 (Additional file 1: Fig. S3d) [35]. We found three predicted YY1 binding sites in the FGF2 promoter from the JASPAR website, and ChIP-qPCR showed that YY1 can bind to the region of − 1336 to − 1172 bp upstream of the transcription start site of FGF2 (Additional file 1: Fig. S3e-g). To further explore the target of YY1 under hypoxia, we performed ChIP-qPCR with YY1 on the promoters of these angiogenic factors. Notably, ChIP-qPCR analysis showed that hypoxia promoted YY1 binding to the promoters of FGF2, rather than other angiogenic factors, including VEGFA, which may be because retinal microglia are not the major cells secreting such angiogenic factors according to previous studies [9, 36–38] (Fig. 5g). Meanwhile, K183R mutation resulted in impaired YY1 binding to FGF2 promoters, indicating that it is regulated by the lactylation level of YY1 (Fig. 5h). A dual-luciferase reporter system was applied to verify the result that YY1 directly promotes the transcription of FGF2 under hypoxia and that mutation at the lactylation site of YY1 resulted in lower transcription ability (Fig. 5i). Meanwhile, we found that intravitreal injection of recombinant FGF2 reversed retinal neovascularization inhibited by microglial depletion (Additional file 1: Fig. S3h). Collectively, our findings suggest that FGF2 released from hypoxic microglia is regulated by the lactylation of YY1 and plays an important role in hypoxia-induced angiogenesis.

### p300 affects the lactylation of YY1 and then regulates angiogenesis in vitro

**Fig. 6:**
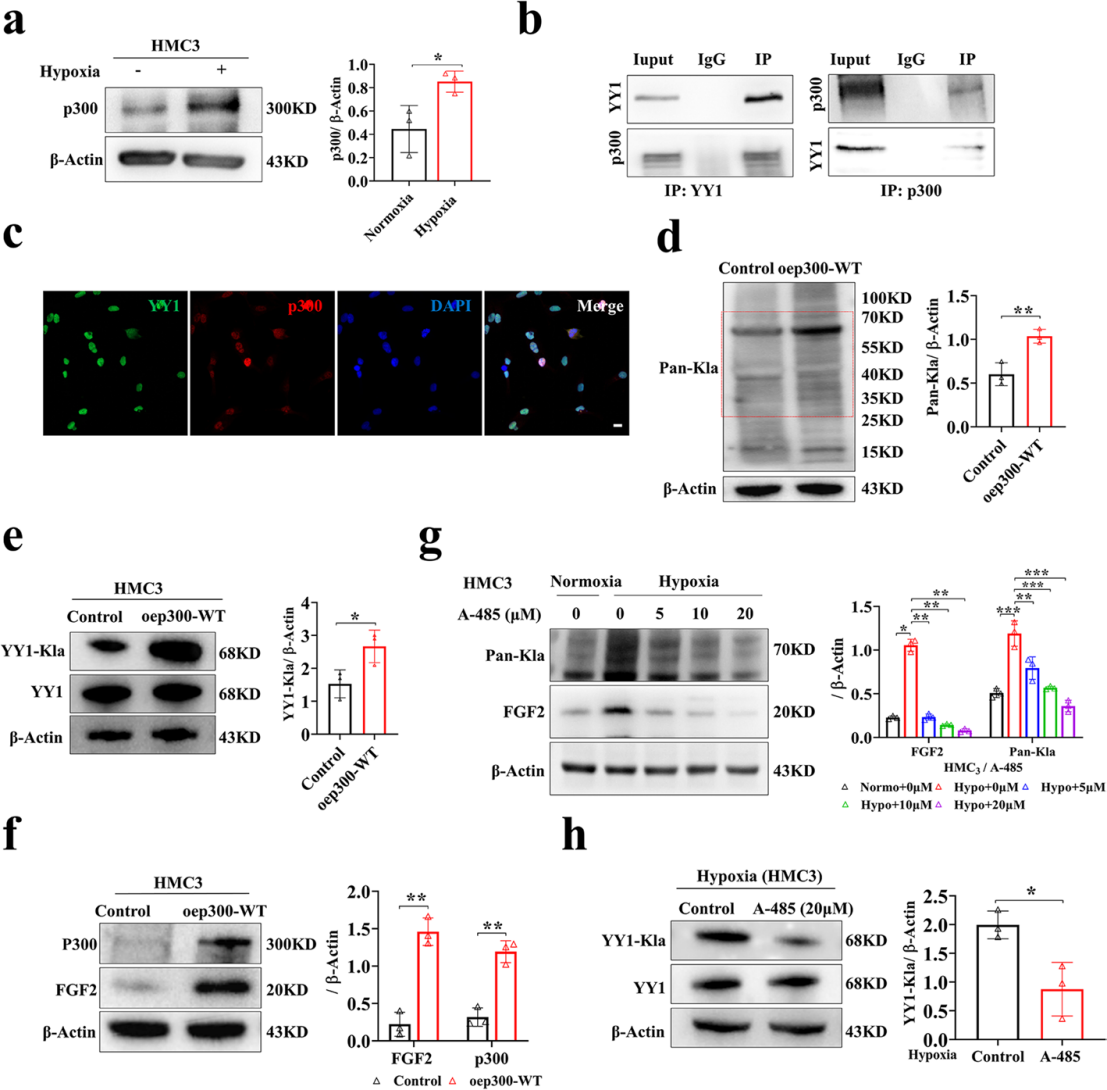
p300 affects the lactylation of YY1 and then regulates angiogenesis in vitro. **a** Quantification of p300 in the HMC3 cells under normoxia or hypoxia for 24 h (*n* = 3 per group). **b** Co-IP analysis of YY1 and p300. **c** Colocalization of YY1 and p300 by double-label immunofluorescence. **d** The Pan-Kla level in the HMC3 cells overexpressing p300 (*n* = 3 per group). **e** Overexpression of p300 increased YY1 lactylation. Lactylation of YY1 in control group, or oep300-WT group was detected by YY1-Kla antibodies (*n* = 3 per group). **f** The expression of FGF2 and p300 in the HMC3 cells overexpressing p300 (*n* = 3 per group). **g** Quantification of FGF2 and Pan-Kla in the HMC3 cells treated with 0, 5, 10, and 20 μM A-485 under hypoxia for 24 h (*n* = 3 per group). **h** Applying A-485 decreased YY1 lactylation. Lactylation of YY1 with 0 μM A-485, or 20 μM A-485 was detected by YY1-Kla antibodies (*n* = 3 per group). *p < 0.05; **p < 0.01; ***p < 0.001

Lysine acylation is a series of universal and evolutionarily conserved PTMs [39]. Lactylation is a new form and may share similar writers and erasers with other lysine acylation types. To identify the regulators responsible for modulating lactylation levels, we detected the expression patterns of known acylation modification writers (Tip60, p300, and PCAF) and erasers (HDAC6 and SIRT1) under normoxic and hypoxic conditions [40, 41]. The results showed that p300, HDAC6, and SIRT1 levels were significantly upregulated and Tip60 levels were slightly upregulated in HMC3 microglia under hypoxia (Fig. 6a and Additional file 1: Fig. S4a). It is worth noting that only p300 bound to the target protein YY1 according to the Co-IP data, and the interaction between p300-YY1 was increased along with hypoxia (Fig. 6b and Additional file 1: Fig. S4b-c). Additionally, we also found that p300 colocalized with YY1 by double-label immunofluorescence (Fig. 6c).

To further explore the role of p300 in regulating lactylation levels, we overexpressed p300 in HMC3 microglia and found that the lactylation level was significantly increased (Fig. 6d). Interestingly, the overexpression of p300 increased the lactylation level of YY1, which was accompanied by the upregulation of FGF2 (Fig. 6e, f). To fully confirm the role of p300, the p300 inhibitor A-485 was used in subsequent experiments. A-485 was proven to be a CoA competitive catalytic inhibitor of p300 and L-lactyl-CoA is indispensable for lactylation [42]. Follow-up studies showed that the lactylation of YY1 was significantly reduced, and the expression level of FGF2, the hypothetical target of transcriptional regulation of YY1, was also downregulated after A-485 treatment (Fig. 6g, h). The lactylation levels of YY1 were verified by IB after IP assays (Additional file 1: Fig. S4d-e). The tube formation, spheroid sprouting, migration, and proliferation abilities of HRMECs cocultured with p300-overexpressing HMC3 microglia were enhanced (Additional file 1: Fig. S4f-i) but weakened in the A-485 group compared with the respective control (Additional file 1: Fig. S4j-m).

### Inhibiting p300 reduces YY1 lactylation of retinal microglia and suppresses angiogenesis in OIR

**Fig. 7:**
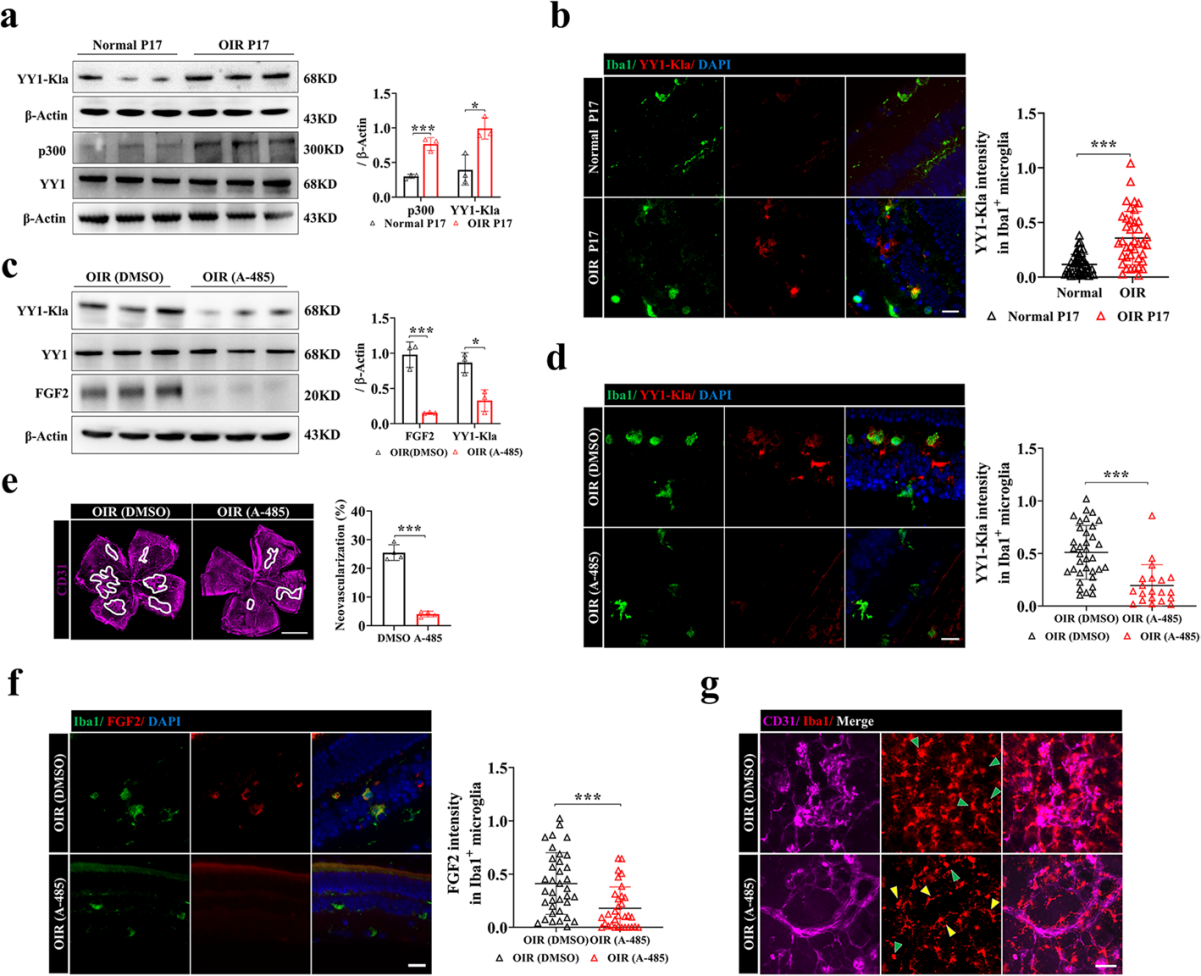
Inhibiting p300 reduces the lactylation of YY1 and suppresses angiogenesis in OIR. **a** p300, YY1, and YY1-Kla in the retinas of normal control (Normal P17) and OIR (OIR P17) mice (*n* = 3 per group). **b** Representative images of YY1-Kla co-stained with microglia (Iba1) in retina of OIR mice (OIR P17) and control mice (Normal P17), with quantification of YY1-Kla intensity (*n* = the number of Iba1^+^ cells); scale bar, 20 μm. **c** Quantification of YY1-Kla and FGF2 in the retinas of OIR mice (OIR P17) treated with A-485 (200 μM, 1 μl/eye) and DMSO (0.1%) were analyzed by Western blotting (*n* = 3 per group). **d** Representative images of YY1-Kla co-stained with microglia (Iba1) in retina of OIR mice treated with A-485 (200 μM, 1 μl/eye) and OIR mice treated with DMSO (0.1%), with quantification of YY1-Kla intensity (*n* = the number of Iba1^+^ cells); scale bar, 20 μm. **e** Confocal images of CD31-stained retinal flat mounts in OIR P17 mice treated with A-485 (200 μM, 1 μl/eye) and DMSO (0.1%). The relative proportion of neovascularization in the retina was calculated and measured by ImageJ (*n* = 4 mice per group); scale bar, 1000 μm. **f** Representative images of FGF2 co-stained with microglia (Iba1) in retina of OIR mice (OIR P17) treated with A-485 (200 μM, 1 μl/eye) and DMSO (0.1%), with quantification of FGF2 intensity (*n* = the number of Iba1^+^ cells); scale bar, 20 μm. **g** Confocal images of CD31 and Iba1-stained retinal flat mounts in OIR P17 mice treated with A-485 (200 μM, 1 μl/eye) and DMSO (0.1%); the yellow arrow denotes the resting-state of microglia, and the green arrow denotes the activated state of microglia; scale bar, 50 μm. *p < 0.05; ***p < 0.001

Knowing the essential proangiogenic role of the p300/YY1 lactylation/FGF2 axis in microglia, we further evaluated the efficacy of antiangiogenic therapy by targeting p300 with A485 in vivo. We found that YY1 lactylation and the expression of p300 were upregulated in the OIR retina (Fig. 7a). Double immunofluorescence staining of Iba1 and YY1-K183la showed upregulated YY1 lactylation in retinal microglia of OIR mice (Fig. 7b). Intravitreal administration of A485 was applied at P14. The Pan-Kla levels were reduced after administration of A485 (Additional file 1: Fig. S5a). Interestingly, A485 treatment decreased YY1 lactylation in retinal microglia (Fig. 7c, d), which was accompanied by the decreased FGF2 expression and alleviated retinal neovascularization (Fig. 7e, f). And the reduced lactylation in microglia may be associated with decreased microglial activation (Fig. 7g). Overall, these results demonstrated that upregulated YY1 lactylation in retinal microglia was associated with retinal neovascularization, indicating that blocking p300-YY1 lactylation-FGF2 signaling may provide a therapeutic paradigm for treating retinal neovascular diseases.

Since murine was born without complete retinal vascular network and the retina is in a relatively hypoxia condition, we wondered whether YY1 lactylation also function in retina developmental angiogenesis. We found hyperlactylation of YY1 in the early stage of retinal development (post-natal day 5). The level of YY1 lactylation decreased at later stage, which indicates that YY1 may also play roles in the physiological angiogenesis (Additional file 1: Fig. S5b). Further study is needed to explore it deeply.

## Discussion

**Fig. 8:**
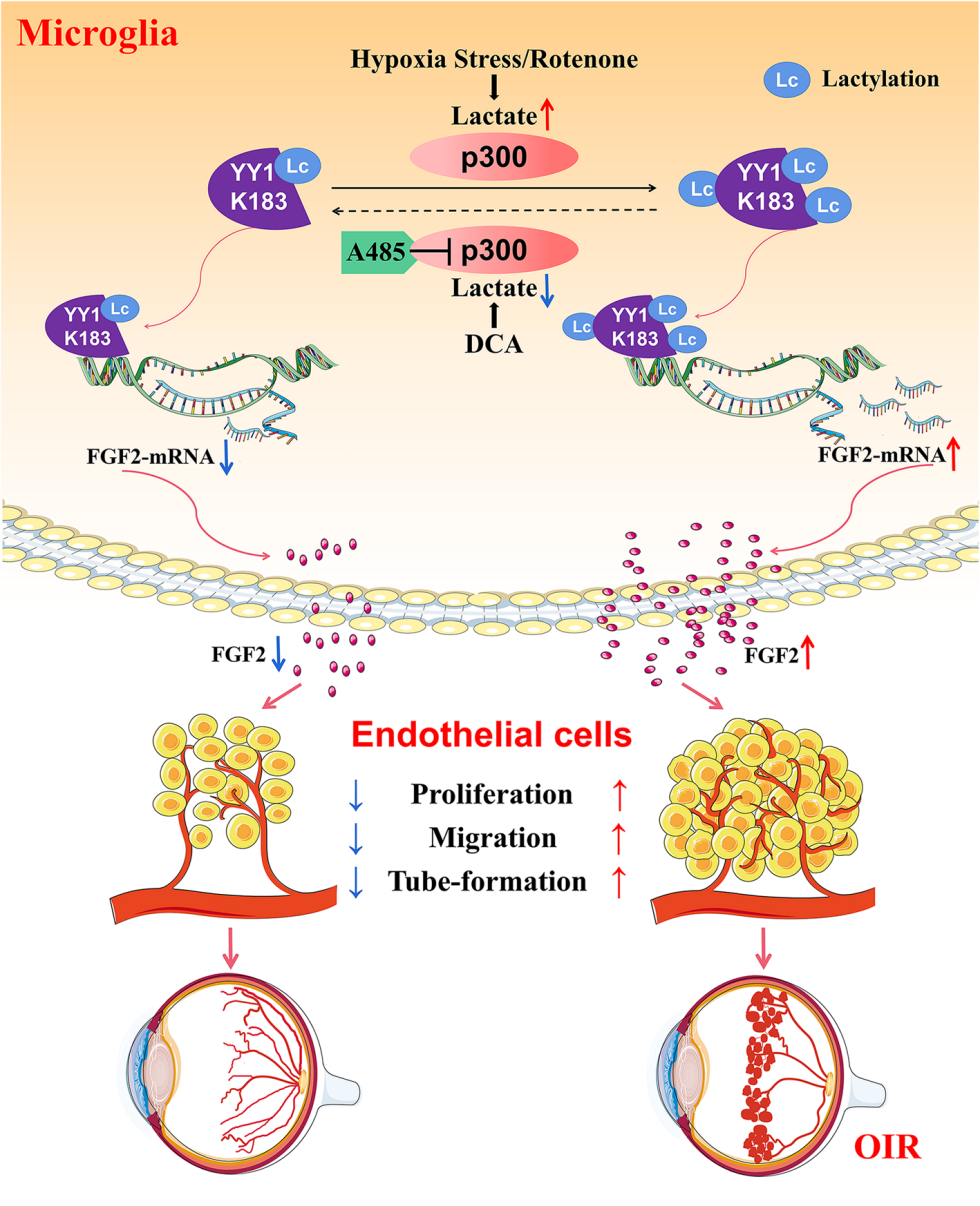
Schematic diagram illustrates the molecular mechanism how YY1 lactylation in microglia contributes to angiogenesis. YY1 lactylation in microglia is upregulated in response to hypoxia. Hyperlactylation of YY1 promotes FGF2 transcription and then contributes to neovascularization

The functional vascular network in the retina is essential for normal vision. Many vision-threatening diseases are caused by proliferative vasculopathies, including ROP, proliferative diabetic retinopathy (PDR), and wet age-related macular degeneration (wAMD) [43, 44]. However, the current therapies are quite limited, indicating the urgent need to elucidate the mechanisms and discover additional or alternative treatments. Anti-VEGF therapy has been established for many years, but many patients are resistant to this treatment, which may be partly because angiogenesis is triggered by multiple angiogenic factors [5]. In this study, we identified the lactate/p300/YY1 lactylation/FGF2 transcription axis as part of an important mechanism underlying microglia-promoted neoangiogenesis. Our findings present a previously undefined regulatory mechanism in which the expression of FGF2 is modulated by YY1(K183) lactylation. Upregulation of YY1 lactylation enhances FGF2 expression and promotes angiogenesis, while mutation at the YY1 lactylation site or reducing YY1 lactylation by A485 reversed this effect (Fig. 8).

Numerous studies have shown that microglial cells contribute to angiogenesis in the brain and retina [45–47]. In pathological angiogenesis, microglial cells that are highly glycolytic are spatially adjacent to endothelial cells (ECs) [46]. Although the important roles of microglia/macrophages in neovascularization have been established for many years, the mechanisms by which they associate directly with nascent vessels and participate in microenvironment interactions remain elusive. Microglia can sense physiological and pathological cues, scan the surrounding microenvironment, and secrete various cytokines and neurotrophic factors when activated [48, 49]. Our data demonstrated that highly glycolytic microglia are regulated by lactylation and induce FGF2 expression, thereby promoting angiogenesis.

YY1 is a multifunctional transcription factor that is able to repress, activate, and initiate transcription relying upon promoter architecture and cellular status. For example, YY1-mediated ZNF322A transcription regulation was eliminated when two YY1 binding sites were depleted at − 462 ~  − 363 in the Del-ZNF322A-pGL4 promoter used for the promoter activity assay [50]. Previous studies have reported that the amino acids between 170 and 200 of YY1 constitute a repressor domain [34, 51, 52]. When the structure of the repressor domain changes, its repression activity is lost. In the cell, YY1 can be transformed into an activating factor only after the N-terminal activation domain is unmasked and exposed under certain conditions [52–55]. Consistent with our conjecture, the lactylation of the lysine at position 183 of YY1 may cause the inhibition mechanism to fail and activate the transcriptional activity of YY1.

The emergence of lactylation has improved our understanding of the function of lactate and its role in a variety of pathophysiological conditions [19, 56]. For example, histone lactylation can directly promote gene transcription and induce the polarization of macrophages from M1 to M2 [18]. Lung myofibroblasts promote the profibrotic activity of macrophages by inducing histone lactylation in the promoters of the profibrotic genes in macrophages [57]. A recent study proposed that Kla in histones is a consequence rather than a cause of macrophage activation, which further proves the multifaceted nature of lactylation [58]. Although some studies have shown that lactylation of histones plays an important role in transcription-related functional regulation, we need to further explore whether lactylation of nonhistone proteins matters and how it works. Our data showed that lactylation at lysine 183 of the nonhistone protein YY1 leads to an increase in the transcriptional ability of YY1, directly upregulates the expression of FGF2, and promotes angiogenesis, supporting the idea that the lactylation of nonhistone proteins can also exert important functions and influence cellular processes.

We speculated that lactylation is similar to the acylation modification that can be directly regulated by some writers and erasers. Our results showed the potential activity of p300 as a Kla writer in cells. Overexpressing p300 resulted in upregulated YY1 lactylation. We inhibited p300 with A485 and observed decreased lactylation levels of YY1 both in vivo and in vitro. Our data suggest that p300 can regulate the lactylation level of YY1 and then affect the development of retinal angiogenesis.

Finally, there are still some limitations in our study. First, although we proved that the YY1 K183R mutation in vitro was responsible for a decreased FGF2 expression in microglia, whether in vivo mutation of the YY1 lactylation site could prevent overvascularization remains unknown and is worthy of investigation. The studies of knock-in or overexpression of WT/delactylated YY1 forms in retinal microglia are needed to better elucidate the role of YY1 lactylation in retinal neovascularization. Second, the clinical data suggested that the lactate content was upregulated in ROP infants, but whether it works by upregulating lactylation in retinal microglia needs further investigation.

## Conclusions

In conclusion, this study revealed that the lactylation level of YY1 in microglia is increased in response to hypoxia. Hyperlactylation of YY1 promotes FGF2 expression through transcriptional activation and further contributes to neovascularization. Furthermore, p300 affects angiogenesis by regulating YY1 lactylation and can be a potential target. These findings expand the field of protein epigenetic regulation and provide potential targets for pathological angiogenesis therapy.

## Methods

### Animals

Male and female C57BL/6 J mice obtained from the Experimental Animal Center of Chongqing Medical University (Chongqing, China) were housed in a specific pathogen-free facility. All protocols were approved by the Ethics Committee of the First Affiliated Hospital of Chongqing Medical University (Number: 2019–101) and conformed to the ARVO Statement for the Use of Animals in Ophthalmic and Vision Research.

### Induction of OIR

OIR is a model of ROP that is characterized by the late stage of destructive pathological angiogenesis. Male and female C57BL/6 J pups were defined as P0 when they were born. The pups and suckling mice received food and water freely with a 12/12 h-day/night cycle in normal air for 7 days (P0-P7). On day P7, healthy pups and suckling mice were placed into a glass oxygen chamber together (Chenxi dianzi; Zhejiang; China), with the oxygen flow controlled to 0.50–0.75 L/min and the oxygen concentration maintained at 75 ± 2%. The animals were kept in a glass oxygen chamber for five consecutive days. At P12, the pups were returned to a normal air environment and continually co-bred with the suckling mice for 5 days. The pups were sacrificed at the age of P17, and the retinas were removed for subsequent experiments.

### Microglial depletion

For microglial depletion, mice were intraperitoneally injected with PLX3397 (MedChemExpress, HY-16749, 1 mg/(kg day)) once a day from P10 to P17. PLX3397 was diluted in 1% dimethyl sulfoxide (DMSO), 45% polyethylene glycol 300 (PEG-300), 5% Tween 80, and saline. After injection, no obvious behavioral or health problems were observed.

### Animal treatment

Male and female C57BL/6 J pups were randomly assigned to the control (0.1% DMSO), rotenone, and DCA groups (*n* = 3 ~ 5 in each group). Rotenone and DCA were freshly prepared every day in a solution of 0.1% DMSO (diluted with PBS) and were administered daily (i.p., rotenone (Rhawn, R006873), 1.5 mg/(kg day); sodium dichloroacetate (i.p., DCA, Rhawn, R052025), 200 mg/(kg day)) from P13 to P16 as previously described [59, 60]. Pups in the control group were administered an equal amount of 0.1% DMSO. One microliter of recombinant murine FGF2 protein (100 ng/ ml, R&D Systems, 3139-FB) was administered by intravitreal injection to the eyes at P14. A485 was diluted in 0.1% DMSO to 200 μM. One microliter of A485 was administered by intravitreal injection to the left eye at P14, and the same amount of 0.1% DMSO was administered to the right eye. All pups were housed under standard laboratory conditions (a 12-h light/dark cycle with an average room temperature of 25 °C).

### Cell lines and cell cultures

HMC3 cells (ATCC; Manassas, VA, USA) were cultured in EMEM containing 10% fetal bovine serum (FBS; Gibco) and 1% penicillin/streptomycin with 5% CO_2_ at 37 °C. HRMECs (Cell Systems; Seattle, America) were cultured in Complete Classic Medium with Serum & CultureBoost (Cell Systems) at 37 °C in 5% CO_2_. All cell lines were authenticated and confirmed to be mycoplasma free using a PCR Mycoplasma Test Kit (Applied Biosystems, 4460623). For coculture experiments, HMC3s were precultured under the corresponding conditions for 24 h (DCA was added at a concentration of 20 mM; rotenone was added at a concentration of 50 nM; A-485 was added at concentrations of 5, 10, and 20 μM according to previous studies) and then cocultured with HRMECs.

### Quantification of lactate levels

The LA Assay Kit (Solarbio, BC2230) was used to determine the content of lactate in retinal tissues and cell lysates. According to the manufacturer’s instructions, the reducing agent solution and reduction reaction color developing solution were reacted for 20 min at 37 °C, then lactate extracted from different treatment groups was transferred into 96-well plates. The lactate content was determined by detecting the absorbance at 570 nm. Each experiment was repeated three times.

### Immunofluorescence

Eyes obtained on day P17 were placed into 4% paraformaldehyde for 2 h. Then the retinas were sheared into flat mounts and blocked with 5% goat serum and 0.4% Triton X-100 for 1 h, followed by incubation with primary antibodies at 4 °C overnight. Then the retinas were washed carefully and incubated with secondary antibody combinations for 1 h. Images were taken by confocal microscopy (Zeiss, Germany). CellSens Dimension software was used to determine the number of microglia (Iba1^+^). Relative fluorescence intensity was measured by ImageJ. The following primary antibodies were used: CD31 (Abcam, ab9498, diluted 1:1000); IBA1 (WAKO, 019–19,741, diluted 1:1000); Pan-Kla (PTM-1401, diluted 1:200);YY1-K183la (PTM, diluted 1:200); YY1 (Proteintech, 66281–1-lg, diluted 1:200); p300 (Santa Cruz, sc48343, diluted 1:200); and Ki67 (Abcam, ab16667, diluted 1:200). The following secondary antibodies were used: Alexa Fluor 488-labelled goat anti-rabbit IgG (H + L) (Beyotime, A0423); Alexa Fluor 488-labelled goat anti-mouse IgG (H + L) (Beyotime, A0428); Cy3-labelled donkey anti-goat IgG (H + L) (Beyotime, A0502); Cy3-labelled goat anti-mouse IgG (H + L) (Beyotime, A0521); Cy3-labelled goat anti-rabbit IgG (H + L) (Beyotime, A0516); Alexa Fluor 647-labeled goat anti-mouse IgG (H + L) (Beyotime, A0473). All secondary antibodies were diluted 1:500.

### Quantitative real-time PCR

RNA was isolated using TRIzol reagent from HMC3 cells (1 × 10^6^ cells) or retinal tissue. cDNA was generated by the RT Master Mix for qPCR (MCE, HY-K0510). Real-time qPCR was performed using SYBR Green qPCR Master Mix (MCE, HY-K0501) with the ABI Prism 7500 system (Applied Biosystems, CA, USA). The specific primers were synthesized by Shanghai Sangon Co. Ltd (Shanghai, China), and the PCR primers employed are listed in Table S2 (Additional file 3: Table S2). All of the reactions were performed at least in triplicate with β-actin as the control.

### Western blotting

Tissue and cell lysates were prepared with RIPA lysis buffer (Beyotime, P0013B), and the protein concentrations were determined using a BCA assay kit (Beyotime, P0012S). The same amount of protein (30 μg) was separated by 8–12% SDS-PAGE and transferred onto PVDF membranes (Millipore, MA, USA). The membranes were blocked with 5% skim milk, followed by incubation with primary antibodies overnight at 4 °C and secondary antibodies for 1 h. The signals were detected using an ECL kit (Advansta, CA, USA) and quantified with ImageJ software. The following primary antibodies were used: Pan-Kla (PTM-1401, diluted 1:1000); YY1-K183la (PTM, diluted 1:500); YY1 (Cell Signaling, 46395S, diluted 1:1000); SIRT1 (Abcam, ab7343, diluted 1:1000); PCAF (Abcam, ab12188, diluted 1:1000); FGF2 (Santa Cruz, sc74412, diluted 1:500); p300 (Santa Cruz, sc48343, diluted 1:500); HDAC6 (Santa Cruz, sc28386, diluted 1:500); TIP60 (Santa Cruz, sc166323, diluted 1:500); β-Actin (Proteintech, 20,536–1-AP, diluted 1:3000); VeriBlot for IP Detection Reagent (HRP) (Abcam, ab131366, diluted 1:2000), a special secondary antibody, was used for IB after IP.

### In vitro lentivirus infection and plasmid transfection

HMC3 cells were seeded in 6-well plates at over 60% confluence and used for lentivirus infection. Synergistic activation mediator (including dCAS9-vp64-Puro and sgRNA-p300 lentivirus) and lentiviruses subcloned with cDNA of Flag-tagged YY1 WT or Flag-tagged YY1 K183R mutant were constructed and purchased from Shanghai Genechem Co, Ltd. According to the manufacturer’s instructions, the lentivirus was added at an MOI of 30, and the medium was replaced 8 h later. After puromycin was added for 3 days, the cells were used for further experiments. The plasmids used for luciferase activity assays were constructed and purchased from Shanghai Genechem Co., Ltd. and transfected into HMC3 cells by Lipofectamine™ 2000 (Invitrogen, 11,668,019).

### Pan antibody-based PTM enrichment

HMC3 cells were treated with hypoxia or normoxia for 24 h. To enrich Kla modified peptides, the tryptic peptides were dissolved in NETN buffer (1 mM EDTA, 50 mM Tris–HCl, 100 mM NaCl, 0.5% NP-40, pH 8.0), followed by incubation with prewashed beads (PTM Bio) at 4 °C for 12 h. Trifluoroacetic acid (0.1%) was used to elute the bound peptides. The eluted peptides were vacuum-dried and desalted with C18 ZipTips (Millipore) for LC‒MS/MS analysis.

### LC‒MS/MS analysis and database search

LC‒MS/MS analysis was supported by Jingjie PTM BioLabs (Hangzhou, China). In brief, the tryptic peptides were dissolved in solvent A (0.1% formic acid, 2% acetonitrile/in water) and separated by a homemade reversed-phase analytical column (25 cm length, 100 μm i.d.) on a nanoElute UHPLC system (Bruker Daltonics). After being subjected to a capillary source, the peptides were subjected to timsTOF Pro (Bruker Daltonics) mass spectrometry for further analysis in parallel accumulation serial fragmentation (PASEF) mode. The MS/MS data were processed by the MaxQuant search engine (v.1.6.6.0). Tandem mass spectra were searched using the human SwissProt database (20,366 entries) and reverse decoy database. FDR < 1%. The relative quantitative values of modified peptides in different samples were obtained by centralizing the signal intensity values in different samples. After filtering lysine lactylation sites (localization probability > 0.75), the relative quantitative values of each sample were obtained by two experiments. All the ratios of quantified lysine lactylation peptides were normalized according to their corresponding protein expression levels. The protein pathways were annotated using the KEGG database. The STRING database was used to identify protein–protein interactions of the DELPs.

### Coimmunoprecipitation (Co-IP)

An immunoprecipitation kit (Abcam, ab206996) was used for immunoprecipitation of the corresponding proteins. Following the manufacturer’s instructions, the cell lysates were incubated with antibodies for 12 h at 4 °C. Forty microliters of protein A/G beads was prewashed and then incubated with beads for another 2 h. After extensive washing, bound proteins were processed by Western blotting using the corresponding antibodies.

### ChIP assay

The ChIP assay was performed using a SimpleChIP Plus Enzymatic Chromatin IP Kit (CST, 9004S). Briefly, 1 × 10^7^ HMC3 cells after fixation were resuspended in ChIP buffer containing 1 × Protease Inhibitor Cocktail (CST). Then, micrococcal nuclease was used to shear chromatin DNA into fragments ranging between 150 and 900 bp. The DNA fragments were immunoprecipitated with 10 µl precleared protein A/G agarose beads overnight at 4 °C with anti-YY1 (CST, 46395S) or anti-IgG (CST, 3900) antibodies. YY1 occupancy on the FGF2 promoter was examined using RT-quantitative PCR analysis. The ChIP‒qPCR primers employed are listed in Supplementary Table 2 (Additional file 3: Table S2).

### Luciferase activity assays

The FGF2 promoter region was cloned into the luciferase reporter vector. YY1 WT and K183R HMC3 cells and control HMC3 cells were seeded in 96-well plates and cotransfected with the constructed plasmids. After 24 h in normoxia, the cells were transferred to hypoxia for another 24 h. A dual-luciferase reporter gene assay kit (Promega, E2920) was used to detect the luciferase activities. Renilla luciferase activity was used to normalize reporter gene activities.

### HRMECs tube formation assay

Endothelial cell tube formation ability was evaluated by Matrigel purchased from Corning. Briefly, 50 µl of Matrigel was placed into each well of 96-well plates and incubated at 37 °C for 40 min in a 5% CO_2_ incubator. Microglia were exposed to different treatments for 24 h. A total of 2.0 × 10^4^ HRMECs were seeded per well on Matrigel and cocultured with pretreated microglia. Tube formation at the indicated times of 12 and 20 h was assessed by microscopy. We captured 3 random images per well and Image-Pro Plus software was used to measure the length of the tubes. Each experiment was repeated three times.

### Spheroid-based sprouting angiogenesis assay

The HRMEC capillary sprouting assay was performed following established protocols [61]. HRMECs were added to the fresh conical tube and treated with methocel stock solution improving spheroid formation then incubated in a humidified cell culture incubator set at 37 °C and 5% CO_2_ for 24 h. Spheroids were embedded in the 24-well plate, cultured with collagen medium containing 20% FBS in a humidified cell culture and incubated at 37 °C for 24 h. Photographs of spheroids were taken with a microscope (Olympus IX73, OLYMPUS, Tokyo, Japan). Photographs were used to quantify the total sprout length with FIJI by measuring the cumulative sprout length of all sprouts per spheroid.

### HRMEC migration assay

The migration of HRMECs was assayed using 24-well Transwell chambers with 8-μm pore size filters (Corning, 3422). Briefly, microglia were seeded in the bottom chamber and pretreated for 24 h. Then 1.5 × 10^4^/ml HRMECs were placed into the upper chamber and cocultured at 37 °C under 5% CO_2_ for 24 h. After fixation with 4% paraformaldehyde for 30 min, HRMECs were stained with 1% crystal violet. The number of the cells migrating to the bottom side of the filter was counted after wiping away the cells on the upper surface. Three images were captured per well under a microscope screening station (scanR, OLYMPUS, Tokyo, Japan). Each experiment was repeated three times.

### HRMEC proliferation assay

The proliferation of HMRECs was assayed using 24-well Transwell chambers with 0.3-μm pore size filters (Corning, 3413). Briefly, microglia were exposed to different treatments for 24 h. Then 2.0 × 10^4^/ml HRMECs were placed in the bottom chamber and cocultured with the pretreated microglia for 24 h at 37 °C under 5% CO_2_. After fixation with 4% paraformaldehyde for 30 min, the proliferating cells were stained with Ki67. Three images were captured per well, and each experiment was repeated three times.

### Clinical retrospective study

The clinical retrospective study included 121 preterm infants (28 ~ 33 weeks of gestational age) who were admitted to the Children’s Hospital of Chongqing Medical University between June 2019 and July 2021 (matching criteria: sex, age, weight, gestational age, birth weight, multiple birth, ethnic group, and multiple regression analysis was used to exclude the impact of these criteria) (Additional file 2: Table S1). We excluded preterm infants who were suffering extremely serious diseases, such as multiple organ failure, cardiac arrest, septicemia, and respiratory diseases that may directly influence the concentration of lactate in blood, including acute respiratory distress syndrome, respiratory failure, and severe pneumonia. ROP diagnostic examinations were performed using indirect ophthalmoscopy. The levels of blood lactate were measured by arterial blood gas analysis prior to commencing artificial ventilation treatment. All protocols were approved by the Ethics Committee of the Children’s Hospital of Chongqing Medical University (Number: 2020–289).

### Statistical analysis

All data are presented as the mean ± SD and were analyzed by SPSS 20.0 software (IBM, Chicago, IL, USA). The normal distribution of the data was confirmed by the Shapiro‒Wilk and Kolmogorov–Smirnov tests. Student’s *t* test and Mann–Whitney *U* test were used to compare differences between two groups, and one-way ANOVA followed by a Bonferroni post hoc test was used for three or more groups. Clinical data analysis was conducted with the Mann–Whitney *U* test, Fisher’s exact test, or *χ*^2^-test for various parameters. The differences at *p* < 0.05 were considered statistically significant.

## Supplementary Information

**Additional file 1: Fig. S1.** Experimental flowchart. **Fig. S2.** Sequencing results and transfection efficiency. **Fig. S3.** FGF2 is regulated by YY1 lactylation. **Fig. S4.** Overexpressing p300 enhances endothelial functions, whereas inhibiting p300 attenuates endothelial functions. **Fig. S5.** YY1-Kla is important for retinal angiogenesis.

**Additional file 2: Table S1.** Clinical characteristics data.

**Additional file 3: Table S2.** Primers used in this study.

**Additional file 4.** Uncropped blot images.

**Additional file 5.** Review history.

## Data Availability

The raw and processed proteomic and lactylome data are available in the Proteomics Identification Database with accession number PXD028737 [62]. All microscopy images are available at Figshare repositor [63]. All the other data generated in this study are included in the article and the Additional files.

## References

[1] Hellstrom A, Smith LE, Dammann O (2013). Retinopathy of prematurity. Lancet.

[2] Hartnett ME, Penn JS (2012). Mechanisms and management of retinopathy of prematurity. N Engl J Med.

[3] Kandasamy Y, Hartley L, Rudd D, Smith R (2017). The association between systemic vascular endothelial growth factor and retinopathy of prematurity in premature infants: a systematic review. Br J Ophthalmol.

[4] Kim YH, Chung IY, Choi MY, Kim YS, Lee JH, Park CH, Kang SS, Roh GS, Choi WS, Yoo JM, Cho GJ (2007). Triamcinolone suppresses retinal vascular pathology via a potent interruption of proinflammatory signal-regulated activation of VEGF during a relative hypoxia. Neurobiol Dis.

[5] Yang S, Zhao J, Sun X (2016). Resistance to anti-VEGF therapy in neovascular age-related macular degeneration: a comprehensive review. Drug Des Devel Ther.

[6] Chan-Ling T, Gole GA, Quinn GE, Adamson SJ, Darlow BA (2018). Pathophysiology, screening and treatment of ROP: A multi-disciplinary perspective. Prog Retin Eye Res.

[7] Agrawal S, Joshi M, Christoforidis JB (2013). Vitreous inflammation associated with intravitreal anti-VEGF pharmacotherapy. Mediators Inflamm.

[8] Stitt AW, Curtis TM, Chen M, Medina RJ, McKay GJ, Jenkins A, Gardiner TA, Lyons TJ, Hammes HP, Simo R, Lois N (2016). The progress in understanding and treatment of diabetic retinopathy. Prog Retin Eye Res.

[9] He C, Liu Y, Huang Z, Yang Z, Zhou T, Liu S, Hao Z, Wang J, Feng Q, Liu Y (2021). A specific RIP3(+) subpopulation of microglia promotes retinopathy through a hypoxia-triggered necroptotic mechanism. Proc Natl Acad Sci U S A.

[10] Li J, Yu S, Lu X, Cui K, Tang X, Xu Y, Liang X (2021). The phase changes of M1/M2 phenotype of microglia/macrophage following oxygen-induced retinopathy in mice. Inflamm Res.

[11] Xu W, Hu Z, Lv Y, Dou G, Zhang Z, Wang H, Wang Y (2018). Microglial density determines the appearance of pathological neovascular tufts in oxygen-induced retinopathy. Cell Tissue Res.

[12] DudvarskiStankovic N, Teodorczyk M, Ploen R, Zipp F, Schmidt MHH (2016). Microglia-blood vessel interactions: a double-edged sword in brain pathologies. Acta Neuropathol.

[13] Boeck M, Thien A, Wolf J, Hagemeyer N, Laich Y, Yusuf D, Backofen R, Zhang P, Boneva S, Stahl A (2020). Temporospatial distribution and transcriptional profile of retinal microglia in the oxygen-induced retinopathy mouse model. Glia.

[14] Pucino V, Certo M, Bulusu V, Cucchi D, Goldmann K, Pontarini E, Haas R, Smith J, Headland SE, Blighe K (2019). Lactate buildup at the site of chronic inflammation promotes disease by inducing CD4(+) T cell metabolic rewiring. Cell Metab.

[15] Zhang J, Muri J, Fitzgerald G, Gorski T, Gianni-Barrera R, Masschelein E, D'Hulst G, Gilardoni P, Turiel G, Fan Z (2020). Endothelial lactate controls muscle regeneration from ischemia by inducing M2-like macrophage polarization. Cell Metab.

[16] Lee DC, Sohn HA, Park ZY, Oh S, Kang YK, Lee KM, Kang M, Jang YJ, Yang SJ, Hong YK (2015). A lactate-induced response to hypoxia. Cell.

[17] Rabinowitz JD, Enerback S (2020). Lactate: the ugly duckling of energy metabolism. Nat Metab.

[18] Zhang D, Tang Z, Huang H, Zhou G, Cui C, Weng Y, Liu W, Kim S, Lee S, Perez-Neut M (2019). Metabolic regulation of gene expression by histone lactylation. Nature.

[19] Yu J, Chai P, Xie M, Ge S, Ruan J, Fan X, Jia R (2021). Histone lactylation drives oncogenesis by facilitating m(6)A reader protein YTHDF2 expression in ocular melanoma. Genome Biol.

[20] Irizarry-Caro RA, McDaniel MM, Overcast GR, Jain VG, Troutman TD, Pasare C (2020). TLR signaling adapter BCAP regulates inflammatory to reparatory macrophage transition by promoting histone lactylation. Proc Natl Acad Sci U S A.

[21] Lu Y, Brommer B, Tian X, Krishnan A, Meer M, Wang C, Vera DL, Zeng Q, Yu D, Bonkowski MS (2020). Reprogramming to recover youthful epigenetic information and restore vision. Nature.

[22] Elmore MR, Najafi AR, Koike MA, Dagher NN, Spangenberg EE, Rice RA, Kitazawa M, Matusow B, Nguyen H, West BL, Green KN (2014). Colony-stimulating factor 1 receptor signaling is necessary for microglia viability, unmasking a microglia progenitor cell in the adult brain. Neuron.

[23] Sosna J, Philipp S, Albay R, Reyes-Ruiz JM, Baglietto-Vargas D, LaFerla FM, Glabe CG (2018). Early long-term administration of the CSF1R inhibitor PLX3397 ablates microglia and reduces accumulation of intraneuronal amyloid, neuritic plaque deposition and pre-fibrillar oligomers in 5XFAD mouse model of Alzheimer's disease. Mol Neurodegener.

[24] Katayama Y, Kawata Y, Moritoh Y, Watanabe M (2022). Dichloroacetate, a pyruvate dehydrogenase kinase inhibitor, ameliorates type 2 diabetes via reduced gluconeogenesis. Heliyon.

[25] Ehinger JK, Piel S, Ford R, Karlsson M, Sjövall F, Frostner E, Morota S, Taylor RW, Turnbull DM, Cornell C (2016). Cell-permeable succinate prodrugs bypass mitochondrial complex I deficiency. Nat Commun.

[26] Yang W, Li Z, Qin R, Wang X, An H, Wang Y, Zhu Y, Liu Y, Cai S, Chen S (2019). YY1 promotes endothelial cell-dependent tumor angiogenesis in hepatocellular carcinoma by transcriptionally activating VEGFA. Front Oncol.

[27] Fang JH, Zhou HC, Zeng C, Yang J, Liu Y, Huang X, Zhang JP, Guan XY, Zhuang SM (2011). MicroRNA-29b suppresses tumor angiogenesis, invasion, and metastasis by regulating matrix metalloproteinase 2 expression. Hepatology.

[28] Gordon-Weeks AN, Lim SY, Yuzhalin AE, Jones K, Markelc B, Kim KJ, Buzzelli JN, Fokas E, Cao Y, Smart S, Muschel R (2017). Neutrophils promote hepatic metastasis growth through fibroblast growth factor 2-dependent angiogenesis in mice. Hepatology.

[29] Kuo TC, Tan CT, Chang YW, Hong CC, Lee WJ, Chen MW, Jeng YM, Chiou J, Yu P, Chen PS (2013). Angiopoietin-like protein 1 suppresses SLUG to inhibit cancer cell motility. J Clin Invest.

[30] Zajac E, Schweighofer B, Kupriyanova TA, Juncker-Jensen A, Minder P, Quigley JP, Deryugina EI (2013). Angiogenic capacity of M1- and M2-polarized macrophages is determined by the levels of TIMP-1 complexed with their secreted proMMP-9. Blood.

[31] Ash D, Sudhahar V, Youn SW, Okur MN, Das A, O'Bryan JP, McMenamin M, Hou Y, Kaplan JH, Fukai T, Ushio-Fukai M (2021). The P-type ATPase transporter ATP7A promotes angiogenesis by limiting autophagic degradation of VEGFR2. Nat Commun.

[32] Dong Z, Santeford A, Ban N, Lee TJ, Smith C, Ornitz DM, Apte RS (2019). FGF2-induced STAT3 activation regulates pathologic neovascularization. Exp Eye Res.

[33] Shi Y, Lee JS, Galvin KM (1997). Everything you have ever wanted to know about Yin Yang 1. Biochim Biophys Acta.

[34] Thomas MJ, Seto E (1999). Unlocking the mechanisms of transcription factor YY1: are chromatin modifying enzymes the key?. Gene.

[35] Mei S, Qin Q, Wu Q, Sun H, Zheng R, Zang C, Zhu M, Wu J, Shi X, Taing L (2017). Cistrome Data Browser: a data portal for ChIP-Seq and chromatin accessibility data in human and mouse. Nucleic Acids Res.

[36] Bai Y, Ma JX, Guo J, Wang J, Zhu M, Chen Y, Le YZ (2009). Muller cell-derived VEGF is a significant contributor to retinal neovascularization. J Pathol.

[37] Le YZ (2017). VEGF production and signaling in Muller glia are critical to modulating vascular function and neuronal integrity in diabetic retinopathy and hypoxic retinal vascular diseases. Vision Res.

[38] Cross MJ, Claesson-Welsh L (2001). FGF and VEGF function in angiogenesis: signalling pathways, biological responses and therapeutic inhibition. Trends Pharmacol Sci.

[39] Figlia G, Willnow P, Teleman AA (2020). Metabolites regulate cell signaling and growth via covalent modification of proteins. Dev Cell.

[40] Zhao S, Zhang X, Li H (2018). Beyond histone acetylation-writing and erasing histone acylations. Curr Opin Struct Biol.

[41] Wang ZA, Cole PA (2020). The chemical biology of reversible lysine post-translational modifications. Cell Chem Biol.

[42] Lasko LM, Jakob CG, Edalji RP, Qiu W, Montgomery D, Digiammarino EL, Hansen TM, Risi RM, Frey R, Manaves V (2017). Discovery of a selective catalytic p300/CBP inhibitor that targets lineage-specific tumours. Nature.

[43] Chen M, Luo C, Zhao J, Devarajan G, Xu H (2019). Immune regulation in the aging retina. Prog Retin Eye Res.

[44] Bradley J, Ju M, Robinson GS (2007). Combination therapy for the treatment of ocular neovascularization. Angiogenesis.

[45] Uemura A, Fruttiger M, D'Amore PA, De Falco S, Joussen AM, Sennlaub F, Brunck LR, Johnson KT, Lambrou GN, Rittenhouse KD, Langmann T (2021). VEGFR1 signaling in retinal angiogenesis and microinflammation. Prog Retin Eye Res.

[46] Liu Z, Xu J, Ma Q, Zhang X, Yang Q, Wang L, Cao Y, Xu Z, Tawfik A, Sun Y (2020). Glycolysis links reciprocal activation of myeloid cells and endothelial cells in the retinal angiogenic niche. Sci Transl Med.

[47] Li Z, Xiao J, Xu X, Li W, Zhong R, Qi L, Chen J, Cui G, Wang S, Zheng Y (2021). M-CSF, IL-6, and TGF-β promote generation of a new subset of tissue repair macrophage for traumatic brain injury recovery. Sci Adv.

[48] Fan W, Huang W, Chen J, Li N, Mao L, Hou S (2022). Retinal microglia: Functions and diseases. Immunology.

[49] Umpierre AD, Wu LJ (2021). How microglia sense and regulate neuronal activity. Glia.

[50] Lin CC, Kuo IY, Wu LT, Kuan WH, Liao SY, Jen J, Yang YE, Tang CW, Chen YR, Wang YC (2020). Dysregulated Kras/YY1/ZNF322A/Shh transcriptional axis enhances neo-angiogenesis to promote lung cancer progression. Theranostics.

[51] Yao YL, Yang WM, Seto E (2001). Regulation of transcription factor YY1 by acetylation and deacetylation. Mol Cell Biol.

[52] Bushmeyer SM, Atchison ML (1998). Identification of YY1 sequences necessary for association with the nuclear matrix and for transcriptional repression functions. J Cell Biochem.

[53] Verheul TCJ, van Hijfte L, Perenthaler E, Barakat TS (2020). The why of YY1: mechanisms of transcriptional regulation by Yin Yang 1. Front Cell Dev Biol.

[54] Austen M, Luscher B, Luscher-Firzlaff JM (1997). Characterization of the transcriptional regulator YY1. The bipartite transactivation domain is independent of interaction with the TATA box-binding protein, transcription factor IIB, TAFII55, or cAMP-responsive element-binding protein (CPB)-binding protein. J Biol Chem.

[55] Shi Y, Seto E, Chang LS, Shenk T (1991). Transcriptional repression by YY1, a human GLI-Kruppel-related protein, and relief of repression by adenovirus E1A protein. Cell.

[56] Xu H, Wu M, Ma X, Huang W, Xu Y (2021). Function and mechanism of novel histone posttranslational modifications in health and disease. Biomed Res Int.

[57] Cui H, Xie N, Banerjee S, Ge J, Jiang D, Dey T, Matthews QL, Liu RM, Liu G (2021). Lung myofibroblasts promote macrophage profibrotic activity through lactate-induced histone lactylation. Am J Respir Cell Mol Biol.

[58] Dichtl S, Lindenthal L, Zeitler L, Behnke K, Schlosser D, Strobl B, Scheller J, El Kasmi KC, Murray PJ (2021). Lactate and IL6 define separable paths of inflammatory metabolic adaptation. Sci Adv.

[59] Zhang D, Li S, Hou L, Jing L, Ruan Z, Peng B, Zhang X, Hong JS, Zhao J, Wang Q (2021). Microglial activation contributes to cognitive impairments in rotenone-induced mouse Parkinson's disease model. J Neuroinflammation.

[60] Anemone A, Consolino L, Conti L, Reineri F, Cavallo F, Aime S, Longo DL (2017). In vivo evaluation of tumour acidosis for assessing the early metabolic response and onset of resistance to dichloroacetate by using magnetic resonance pH imaging. Int J Oncol.

[61] Tetzlaff F, Fischer A (2018). Human endothelial cell spheroid-based sprouting angiogenesis assay in collagen. Bio Protoc.

[62] Wang X, Fan W, Li  N, Ma Y, Yao M, Wang G, He S, Li W, Tan J, Lu Q, Hou S (2023). YY1 lactylation in microglia promotes angiogenesis through transcription activation-mediated upregulation of FGF2.

[63] Wang X, Fan W, Li N, Ma Y, Yao M, Wang G, He S, Li W, Tan J, Lu Q, Hou S (2023). YY1 lactylation in microglia promotes angiogenesis through transcription activation-mediated upregulation of FGF2. Figshare.

