# Supplementary material for: YY1 lactylation in microglia promotes angiogenesis through transcription activation-mediated upregulation of FGF2
Source: Genome Biol. 2023 Apr 21;24:87. doi: 10.1186/s13059-023-02931-y (PMC10120156; doi:10.1186/s13059-023-02931-y)
Supplement: Supplementary file 1 — Additional file 1: Fig. S1. Experimental flowchart. Fig. S2. Sequencing results and transfection efficiency. Fig. S3. FGF2 is regulated by YY1 lactylation. Fig. S4. Overexpressing p300 enhances endothelial functions, whereas inhibiting p300 attenuates endothelial functions. Fig. S5. YY1-Kla is important for retinal angiogenesis. [file 13059_2023_2931_MOESM1_ESM.docx]

Supplementary Materials for

YY1 lactylation in microglia promotes angiogenesis through transcription activation-mediated upregulation of FGF2

Xiaotang Wang, Wei Fan, Na Li, Yan Ma, Mudi Yao, Guoqing Wang, Siyuan He, Wanqian Li, Jun Tan, Qi Lu, and Shengping Hou.

.

Correspondence to: sphou828@163.com

Additional file 1: Figs. S1 to S5 and figure legends.


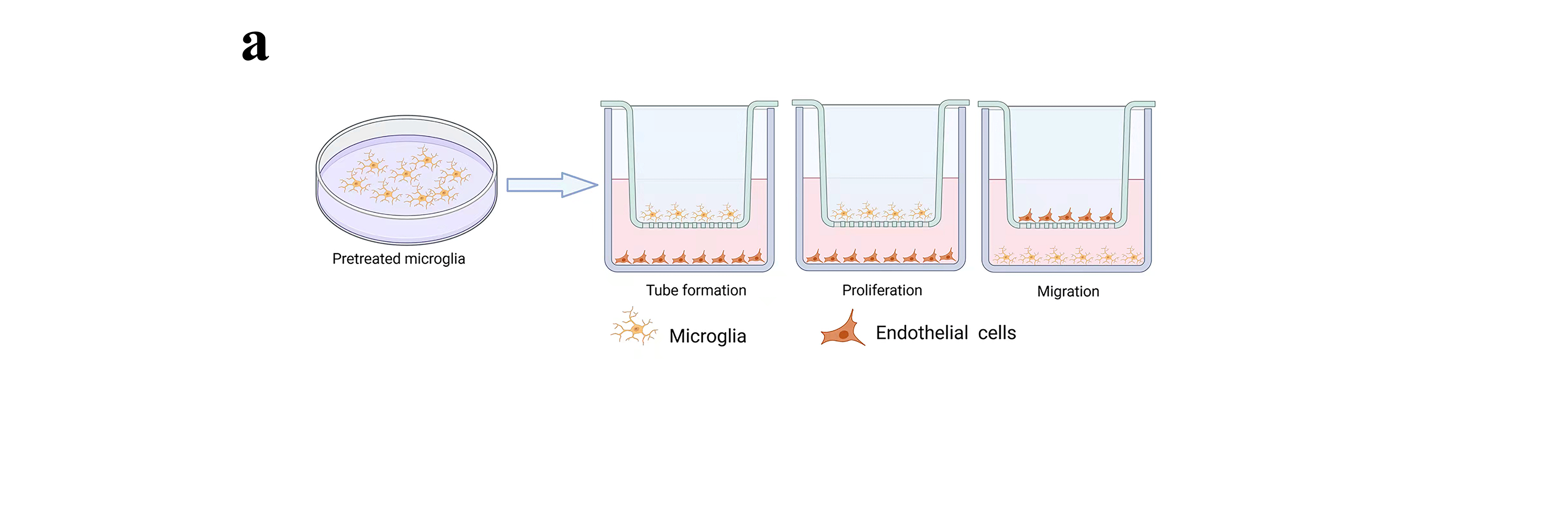


**Fig. S1 Experimental flowchart. (Related to Fig. 3)** **(a)** Microglia are pretreated under various conditions and then cocultured with endothelial cells.


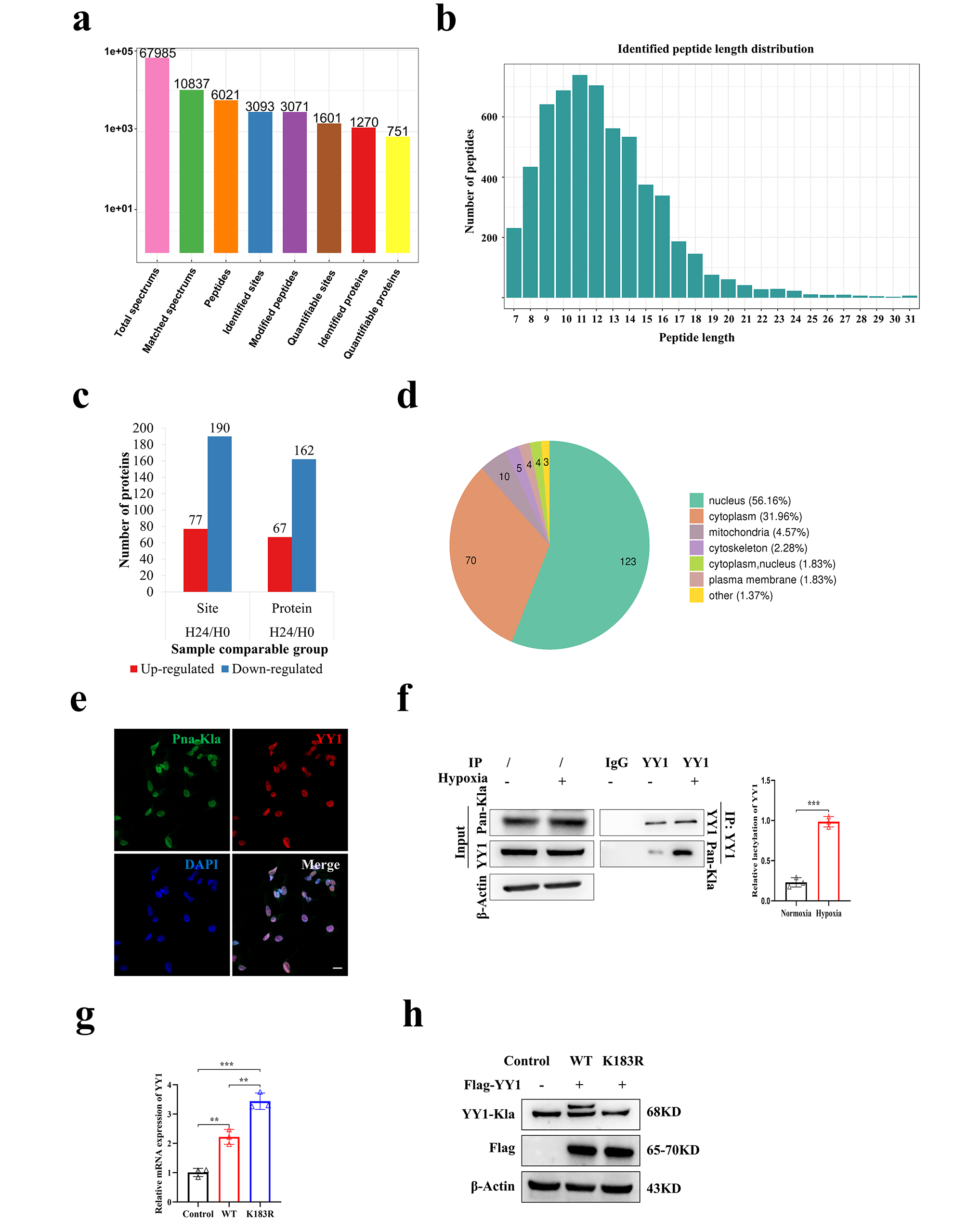


**Fig. S2 Sequencing results and transfection efficiency. (Related to Fig. 4) (a)** The details of the lactylome data. **(b)** The distribution of lactylated protein peptides. **(c)** The number of differentially expressed lactylation proteins (DELPs). **(d)** Subcell classicfy of lactylation differential proteins. **(e)** Co-localization of YY1 and Pan-Kla by double-label immunofluorescence. Scale bar: 20μm. **(f)** Hypoxia increases YY1 lactylation. Lactylation of YY1 in HMC3 cells under normoxia or hypoxia for 24 hrs was detected by Pan anti-Kla antibody (n=3 per group). **(g-h)** Overexpression of flag-tagged WT or K183R YY1 in HMC3 cells, mutants of YY1 at K183 showed lower lactylation (n=3 per group). **p<0.01; ***<0.001


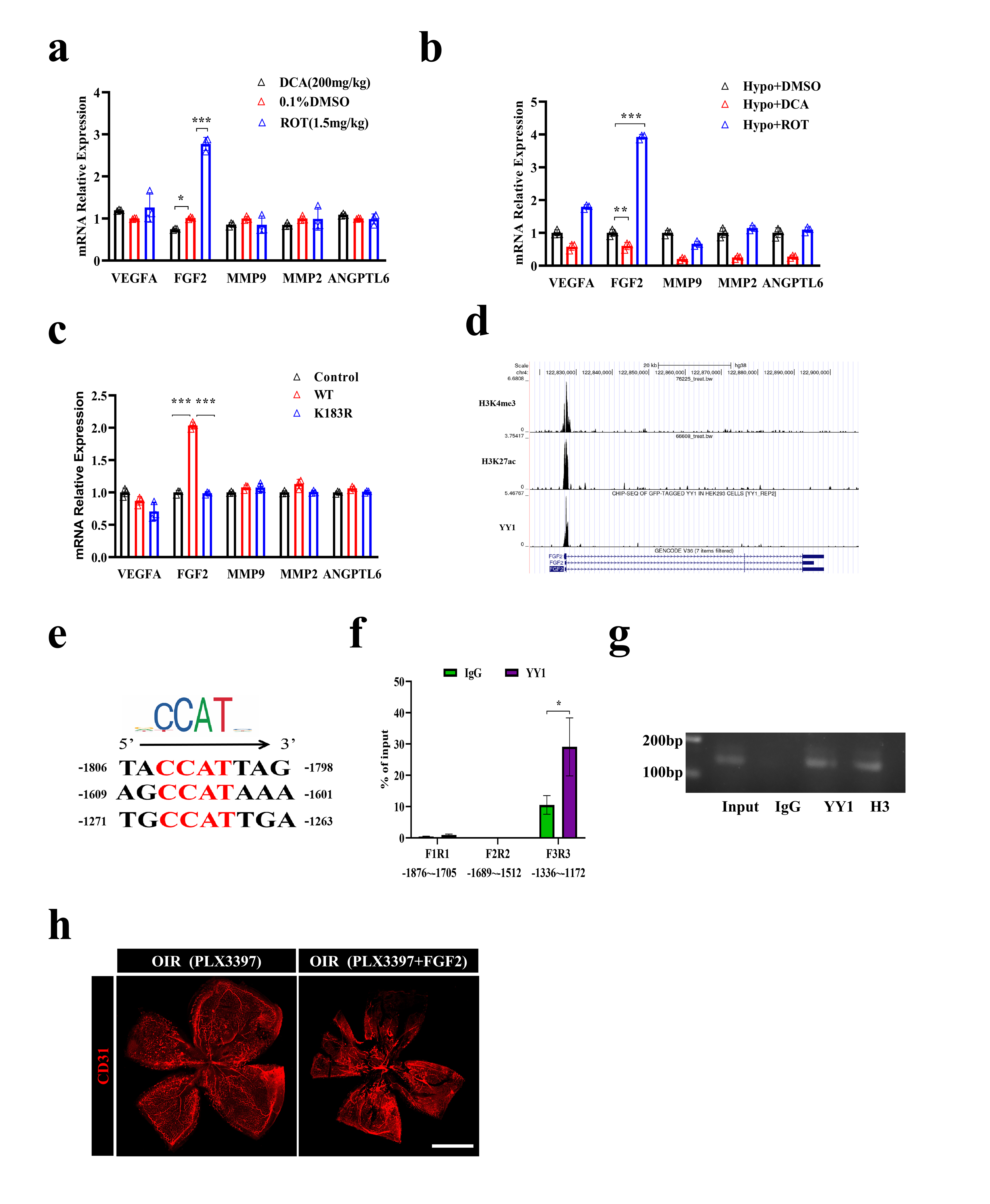


**Fig. S3 FGF2 is regulated by YY1 lactation modification. (Related to Fig. 5)** **(a)** The mRNA expression of VEGFA, FGF2, MMP2, MMP9 and ANGPTL6 in the retina tissue of OIR mice (OIR P17) treated with DCA (200mg/kg), DMSO (0.1%) and Rotenone (n=3 per group). **(b)** The mRNA expression of VEGFA, FGF2, MMP2, MMP9 and ANGPTL6 in the HMC3 cells subjected to hypoxia for 24 hrs+20mM DCA (Hypoxia+DCA), hypoxia for 24 hrs+DMSO (Hypoxia+DMSO) and hypoxia for 24 hrs+50nM rotenone (n=3 per group). **(c)** The mRNA expression of VEGFA, FGF2, MMP2, MMP9 and ANGPTL6 in HMC3 cells exposed to hypoxia for 24 hrs only (Control), hypoxia for 24 hrs+WT transfection (WT) and hypoxia for 24 hrs+K183R transfection (K183R) (n=3 per group). **(d)** Genomic tracks for ChIP-seq around FGF2. **(e)** Potential YY1 binding sites of FGF2 promoter. **(f)** ChIP-qPCR analysis was performed with primers spanning predicted FGF2 promoter sequences (n=3 per group). **(g)** PCR analysis was performed with the primer in the region of -1336 to -1172 bp upstream of the transcription start site of the FGF2. **(h)** Confocal images of CD31-stained retinal flat mounts in OIR P17 mice treated with PLX3397 (OIR (PLX3397)) and PLX3397+FGF2 (OIR (PLX3397+FGF2)); Scale bar, 1000 μm.


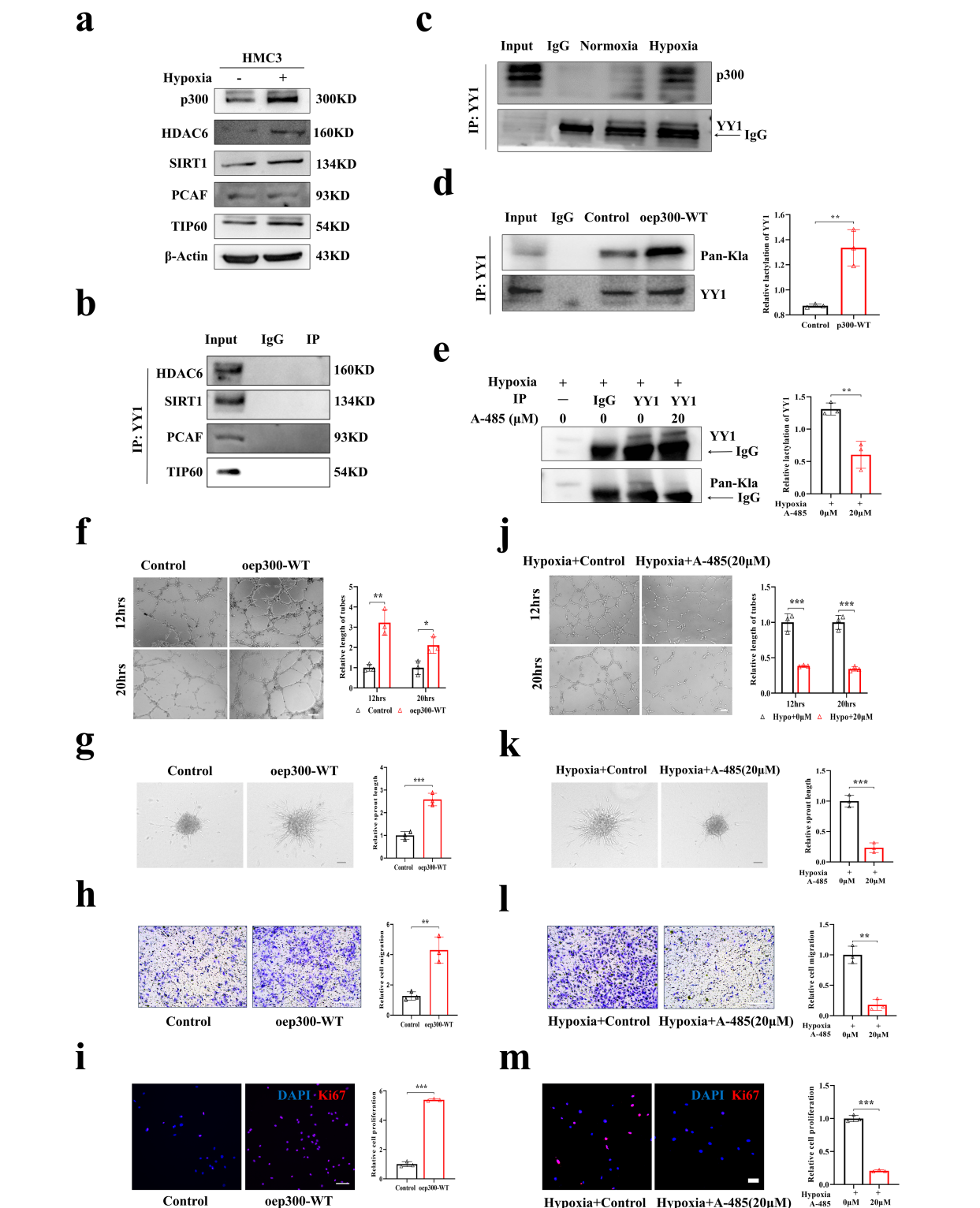


**Fig. S4 Overexpressing p300 enhances endothelial functions, whereas inhibiting p300 attenuates endothelial functions. (Related to Fig. 6)** **(a)** The protein expression of acylation modification writers (Tip60, p300, and PCAF) and erasers (HDAC6 and SIRT1). **(b)** Co-IP analysis of YY1 and the potential writers and erasers. **(c)** Co-IP analysis of YY1 and p300 after treatment with hypoxia and normoxia in HMC3. **(d)** Overexpression of p300 increased YY1 lactylation. Lactylation of YY1 in control group, or oep300-WT group was detected by Pan-Kla antibodies (n=3 per group). **(e)** Applying A-485 decreased YY1 lactylation. Lactylation of YY1 with 0μM A-485, or 20μM A-485 was detected by Pan-Kla antibodies (n=3 per group). **(f-i)** HMC3 cells were transfected with control or p300-overexpression lentivirus. Then HRMECs were cocultured with the pretreated HMC3 cells. The tube formation, spheroid sprouting, migration and proliferation assays were performed as shown in the Methods(n=3 independent experiments, 3 images for each group); **(j-m)** HMC3 cells were pretreated with DMSO or A485 for 24 hrs under hypoxia. Then HRMECs were cocultured with the pretreated HMC3 cells. The tube formation, spheroid sprouting, migration and proliferation assays were performed as shown in the Methods (n=3 independent experiments, 3 images for each group). Scale bars, 100 μm (Fig. **h** and **l**); 50 μm (Fig. **f**, **g**, **i**, **j** and **k**); 25 μm (Fig. **m**). *p<0.05; **p<0.01; ***p<0.001


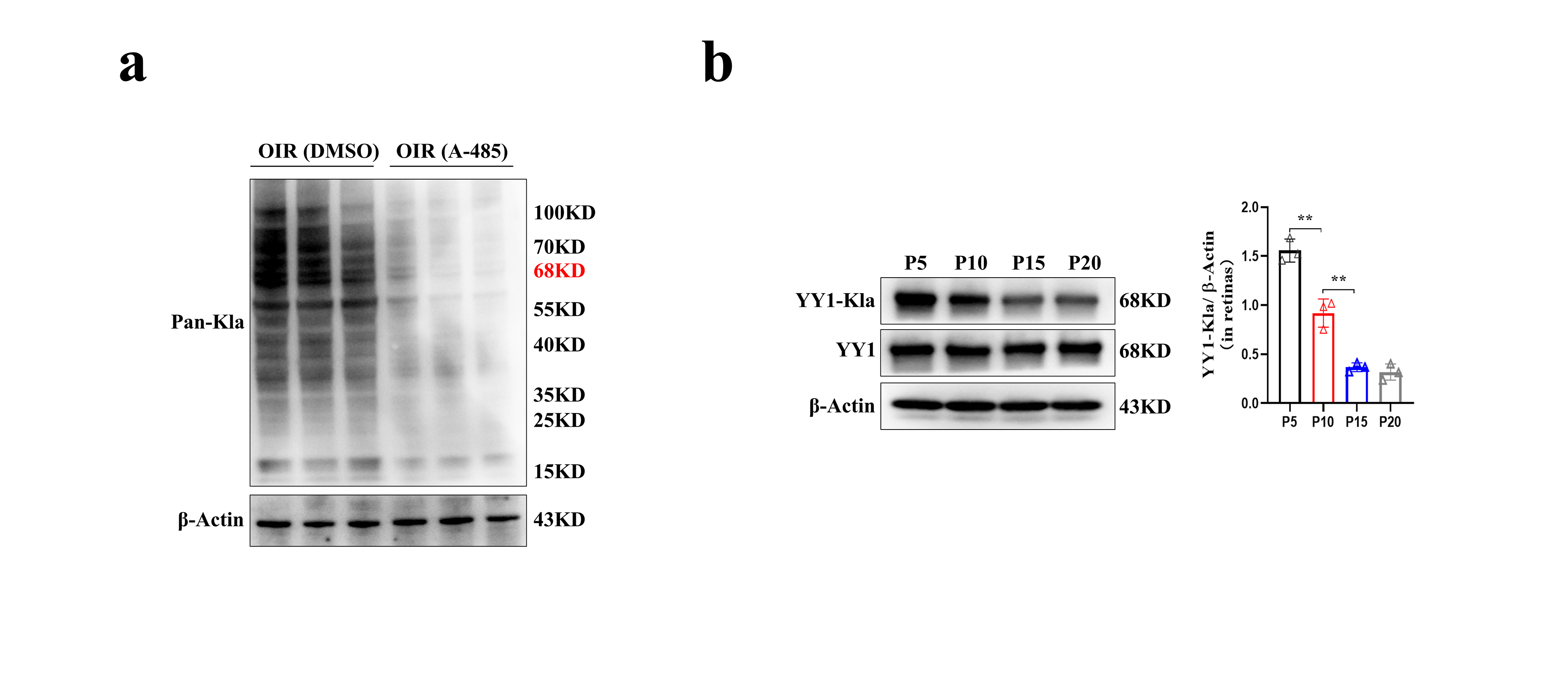


**Fig. S5 YY1-Kla is important for retinal angiogenesis. (Related to Fig. 7) (a)** Quantification of Pan-Kla in the retinas of OIR mice (OIR P17) treated with A-485 (200 μM, 1 μl/eye) and DMSO (0.1%) were analyzed by Western blotting; Blots are representative of three independent experiments. **(b)** YY1-Kla levels in the retinas of normal mice at the time point of P5, P10, P15 and P20 (n=3 per group). **p<0.01
