# Supplementary material for: YY1 lactylation in microglia promotes angiogenesis through transcription activation-mediated upregulation of FGF2
Source: Genome Biol. 2023 Apr 21;24:87. doi: 10.1186/s13059-023-02931-y (PMC10120156; doi:10.1186/s13059-023-02931-y)
Supplement: Supplementary file 2 — Additional file 2: Table S1. Clinical characteristics data. [file 13059_2023_2931_MOESM2_ESM.docx]

| **Table S1. Clinical characteristics data.** | | | |
| --- | --- | --- | --- |
| **Characteristic** | Infants without ROP (n=72) | Infants with ROP (n=49) | P value |
| Age (day)，mean±SD | 54.29±19.30 | 60.41±20.61 | 0.122 |
| Weight (g)，mean±SD | 2695.58±796.47 | 2753.78±696.05 | 0.500 |
| Postmenstrual age at birth (day), mean±SD | 216.21±9.84 | 212.53±11.65 | 0.101 |
| Birthweight (g), mean±SD | 1497.60±282.74 | 1440.76±308.83 | 0.227 |
| **Gender** |  |  |  |
| male,n (%) | 48 (66.7%) | 29 (59.2%) | 0.444 |
| female,n (%) | 24 (33.3%) | 20 (40.8%) |  |
| **Ethnic group** |  |  |  |
| Han，n (%) | 66（91.7%） | 45（91.8%） | 0.349 |
| Miao，n (%) | 3 (4.2%) | 0 |  |
| Tujia，n (%) | 2 (2.8%) | 2（4.1%） |  |
| Buyi，n (%) | 1 (1.3%) | 0 |  |
| Lisu, n (%) | 0 | 1 (2.05%) |  |
| Yao, n (%) | 0 | 1 (2.05%) |  |
| **Multiple birth** |  |  |  |
| 1 | 54 | 40 | 0.506 |
| 2 | 18 | 9 |  |
