# Supplementary material for: YY1 lactylation in microglia promotes angiogenesis through transcription activation-mediated upregulation of FGF2
Source: Genome Biol. 2023 Apr 21;24:87. doi: 10.1186/s13059-023-02931-y (PMC10120156; doi:10.1186/s13059-023-02931-y)
Supplement: Supplementary file 3 — Additional file 3: Table S2. Primers used in this study. [file 13059_2023_2931_MOESM3_ESM.docx]

| **Table S2. Primers used in this study.** | |
| --- | --- |
| **qRT-PCR Primers** | |
| **Human** |  |
| VEGFA | 5′- AGGGCAGAATCATCACGAAGT -3′(forward) |
|  | 5′- AGGGTCTCGATTGGATGGCA -3′(reverse) |
| FGF2 | 5′-AGTGTGTGCTAACCGTTACCT-3′(forward) |
|  | 5′-ACTGCCCAGTTCGTTTCAGTG-3′(reverse) |
| ANGPTL6 | 5′-GGTTCCGGTCCGTCTTGTG-3′(forward) |
|  | 5′-CCCACTCGCAGTTCATACACT-3′(reverse) |
| MMP9 | 5′-TGTACCGCTATGGTTACACTCG-3′(forward) |
|  | 5′-GGCAGGGACAGTTGCTTCT-3′(reverse) |
| MMP2 | 5′-TACAGGATCATTGGCTACACACC-3′(forward) |
|  | 5′-GGTCACATCGCTCCAGACT-3′(reverse) |
| β-actin | 5′-CCTGGCACCCAGCACAAT-3′(forward) |
|  | 5′-GGGCCGGACTCGTCATAC-3′(reverse) |
| **Mouse** |  |
| VEGFA | 5′- TTACTGCTGTACCTCCACC-3′(forward) |
|  | 5′- ACAGGACGGCTTGAAGATG-3′(reverse) |
| FGF2 | 5′-GCGACCCACACGTCAAACTA-3′(forward) |
|  | 5′-TCCCTTGATAGACACAACTCCTC-3′(reverse) |
| ANGPTL6 | 5′-CTGGGCCGTCGTGTAGTAG-3′(forward) |
|  | 5′-CAGTCCTCTAGGAGTATCAGCAG-3′(reverse) |
| MMP9 | 5′-CTGGACAGCCAGACACTAAAG-3′(forward) |
|  | 5′-CTCGCGGCAAGTCTTCAGAG-3′(reverse) |
| MMP2 | 5′-CAAGTTCCCCGGCGATGTC-3′(forward) |
|  | 5′-TTCTGGTCAAGGTCACCTGTC-3 |
| β-actin | 5′-CTACCTCATGAAGATCCTGACC-3′(forward) |
|  | 5′-CACAGCTTCTCTTTGATGTCAC-3′(reverse) |
| **ChIP-qPCR Primers** | |
| FGF2-prom-F1 | TCTGAAGACACACGGACAGG |
| FGF2-prom-R1 | CCCAAAGTTAAGAGGGAAGC |
| FGF2-prom-F2 | TCTCCTGTTTTGTGTAAAACTCACA |
| FGF2-prom-R2 | GGCGCCCTCGCTTGCCTTAC |
| FGF2-prom-F3 | TCGGTAGGCTGAAGTAGGAGA |
| FGF2-prom-R3 | TGAAGCTTTAAAACCAGGCATA |
| VEGFA-prom-F | CGAATGATGGAAAGGGAGGGTTGG |
| VEGFA-prom-R | GGTTTGGTGGAGGTGCTAGGTTAC |
| MMP2-prom-F | ACTGGTGGGTGCTTCCTTTAACATG |
| MMP2-prom-R | AGATGGCAAAGAAAGCGACCTCTG |
| MMP9-prom-F | GGTGTTGCAAAAGGCCAAGGATG |
| MMP9-prom-R | CAGTGGTCAGCCAAGGGAAAGTG |
| ANGPTL6-prom-F | GCAACCCAACTCAATCAGTGACTTC |
| ANGPTL6-prom-R | GGAAGCCAAGATGTGTAGTGTTTGC |
