# Supplementary material for: YY1 lactylation in microglia promotes angiogenesis through transcription activation-mediated upregulation of FGF2
Source: Genome Biol. 2023 Apr 21;24:87. doi: 10.1186/s13059-023-02931-y (PMC10120156; doi:10.1186/s13059-023-02931-y)

Additional file 4. Uncropped western blot images.

Main Fig. 2c

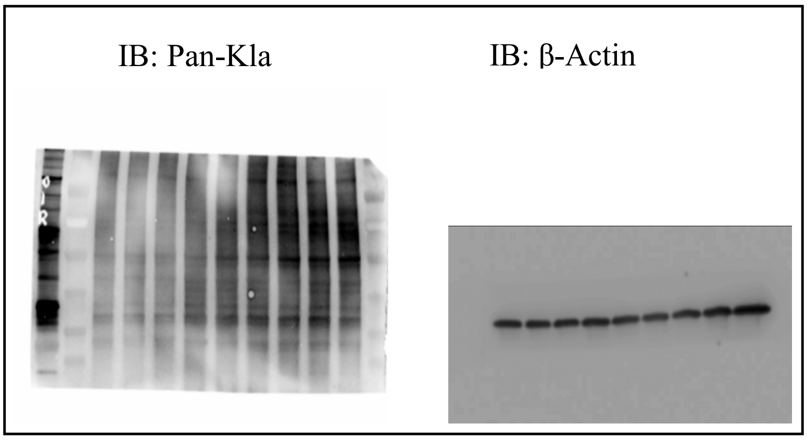

Main Fig. 2e

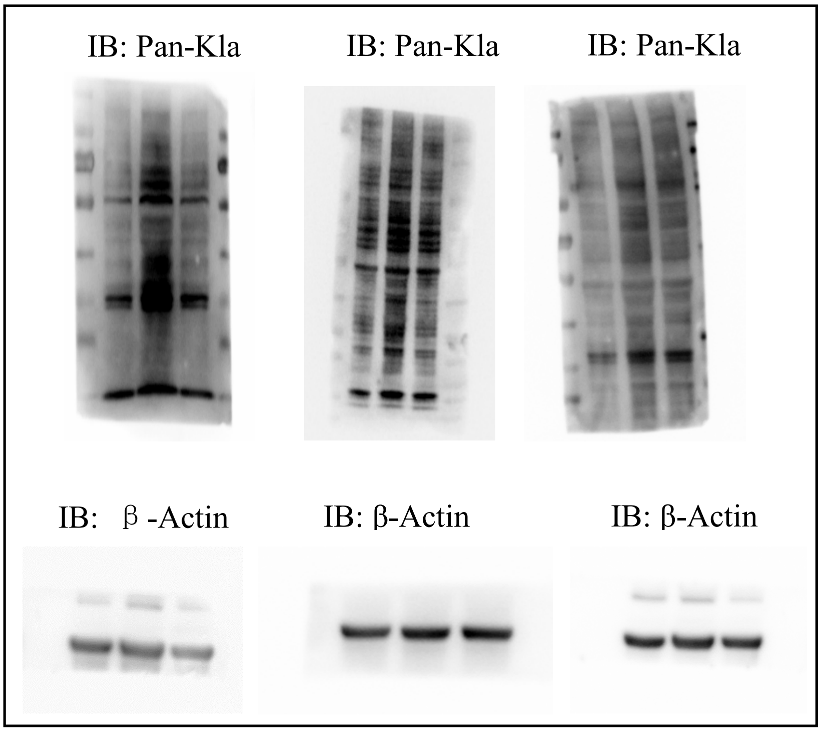

Main Fig. 2g

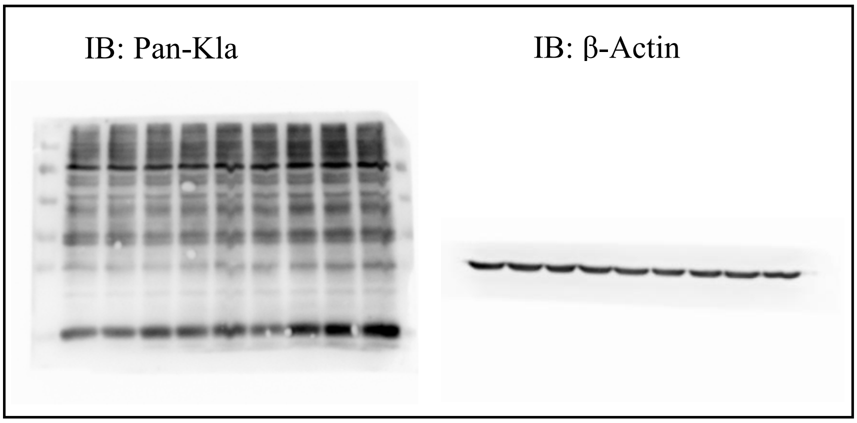

Main Fig. 3b

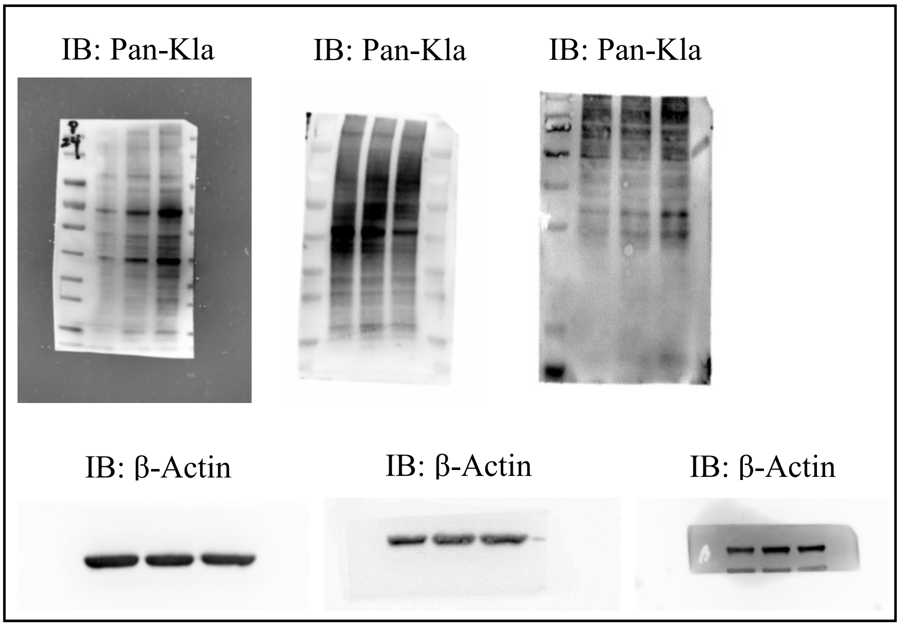

Main Fig. 3h

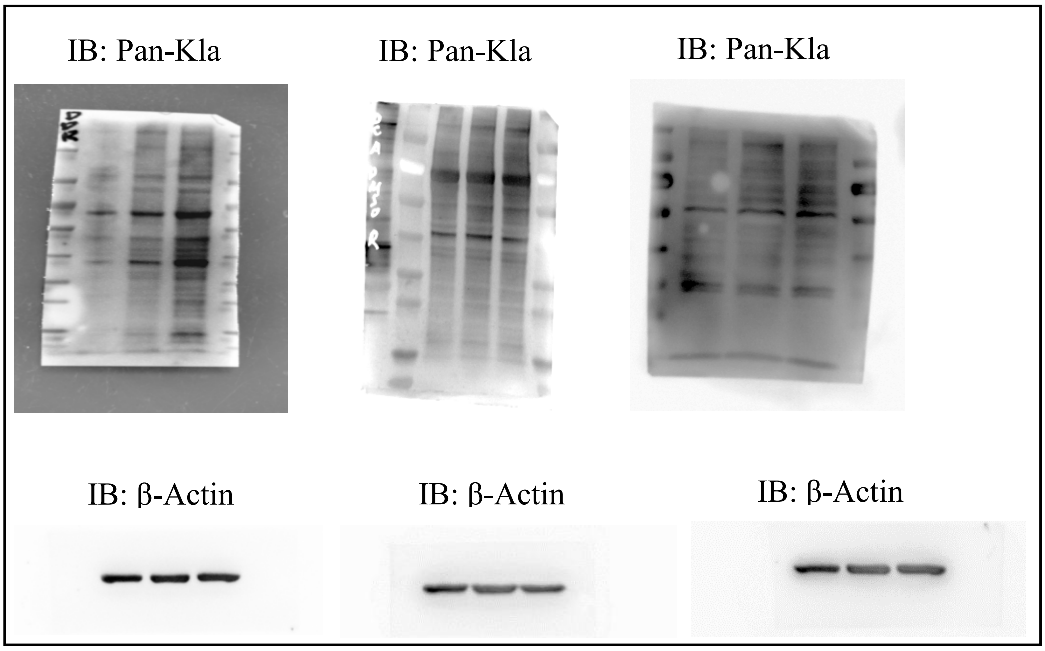

Main Fig. 4e

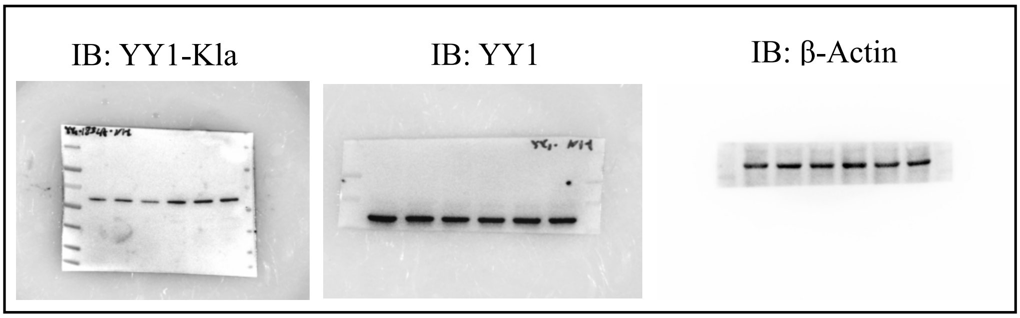

Main Fig. 5c

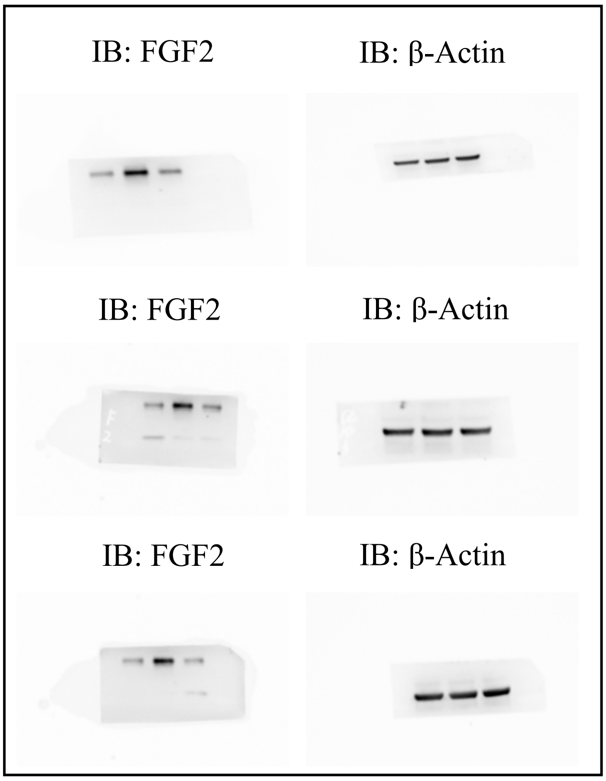

Main Fig. 5d

IB: FGF2

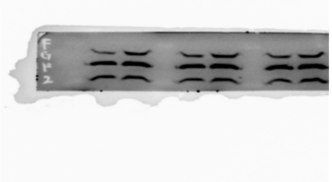

IB: β-Actin

IB: β-Actin

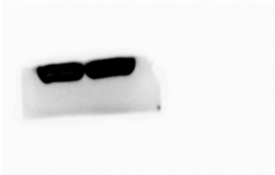

IB: β-Actin

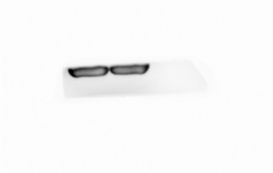

Main Fig. 5f

IB: FGF2

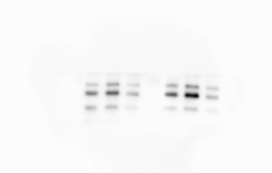

IB: β-Actin

IB: β-Actin

IB: FGF2

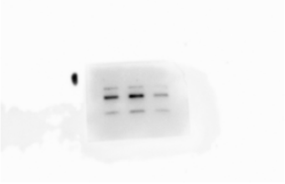

IB: β-Actin

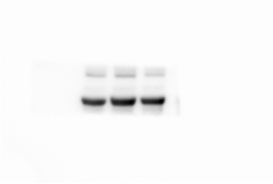

Main Fig. 6a

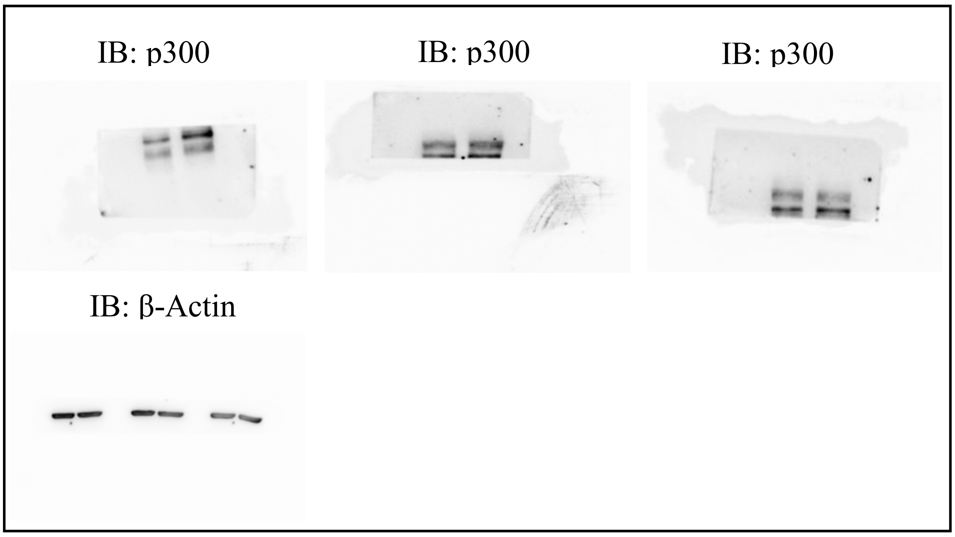

Main Fig. 6b

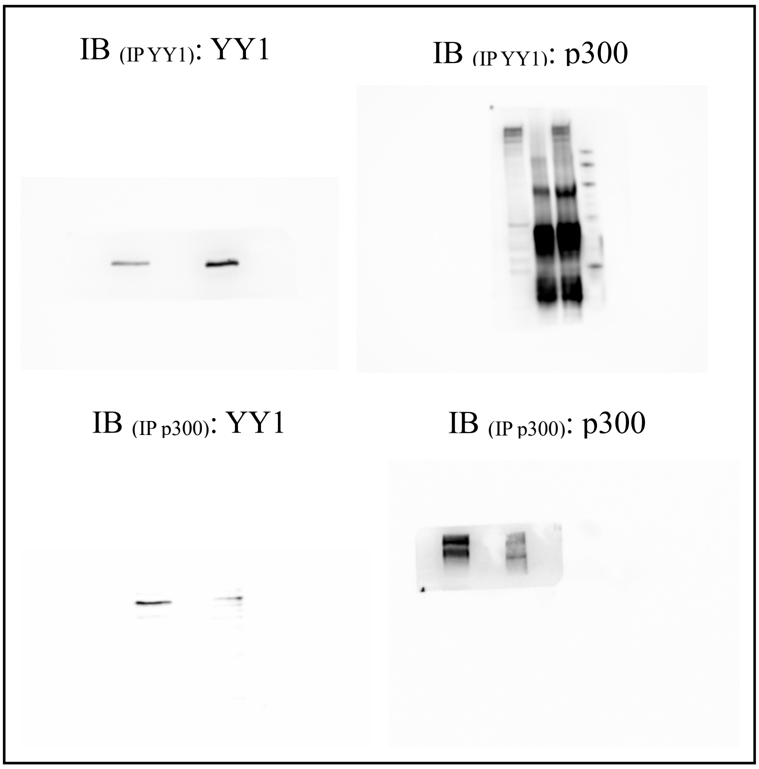

Main Fig. 6d

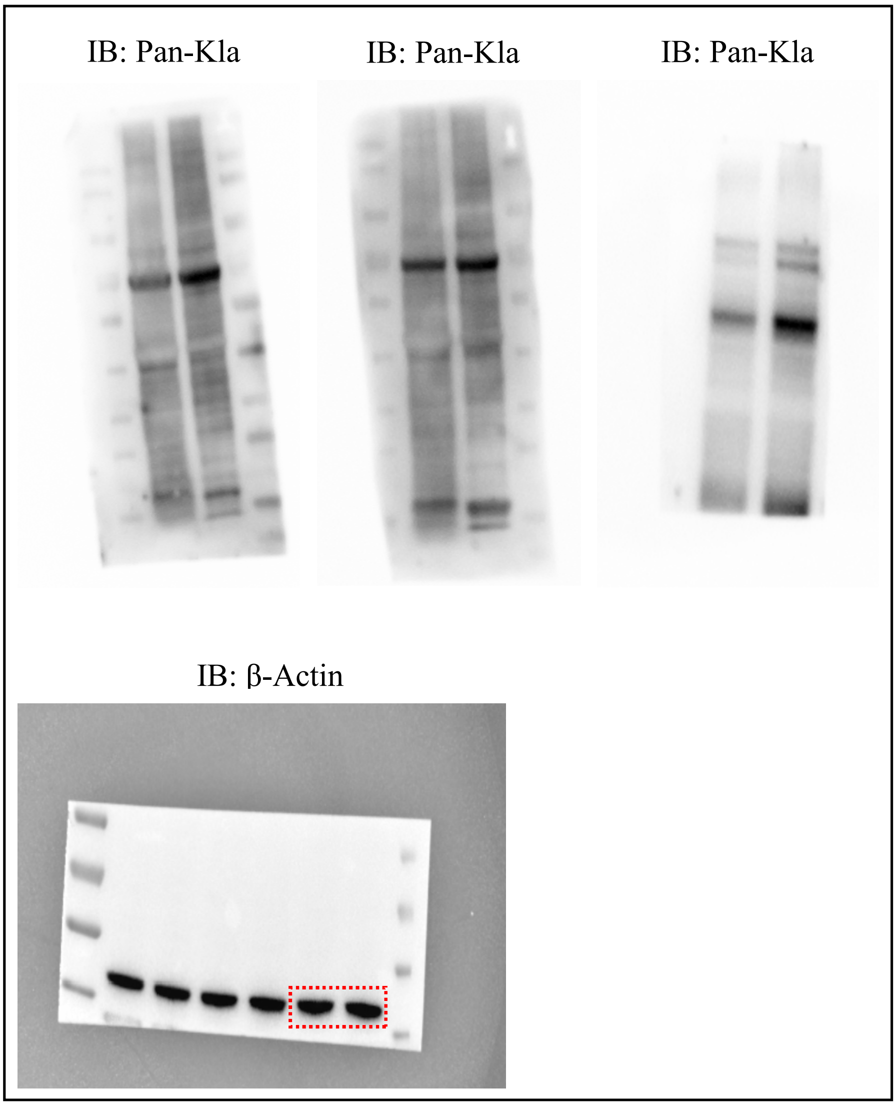

Main Fig. 6e

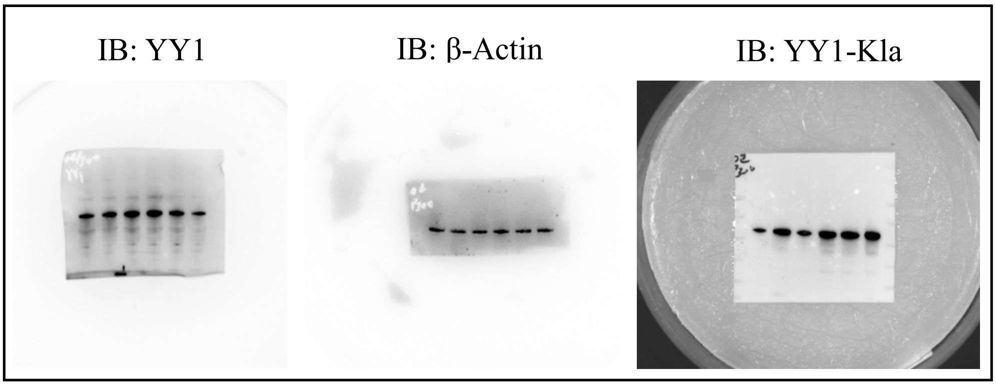

Main Fig. 6f

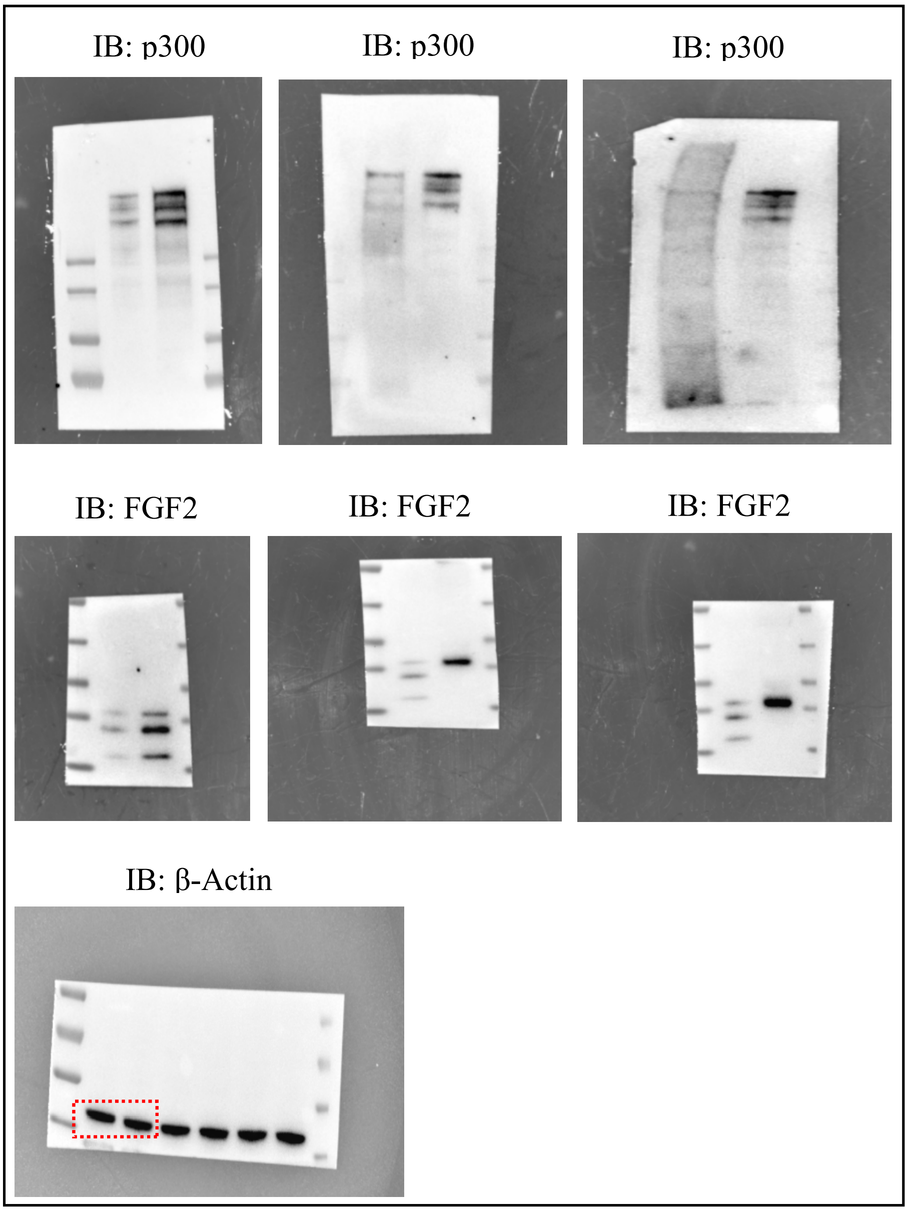

Main Fig. 6g

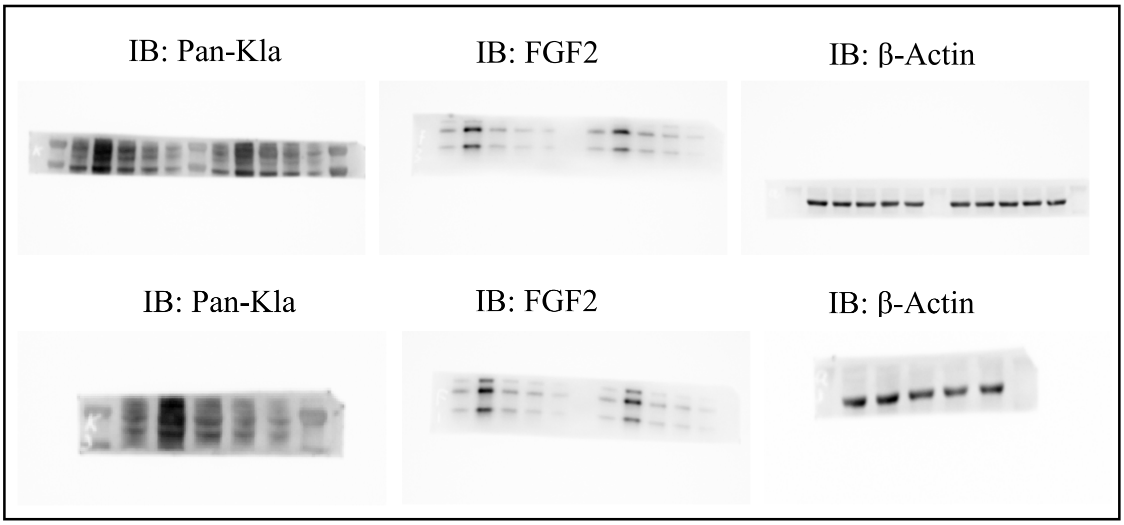

Main Fig. 6h

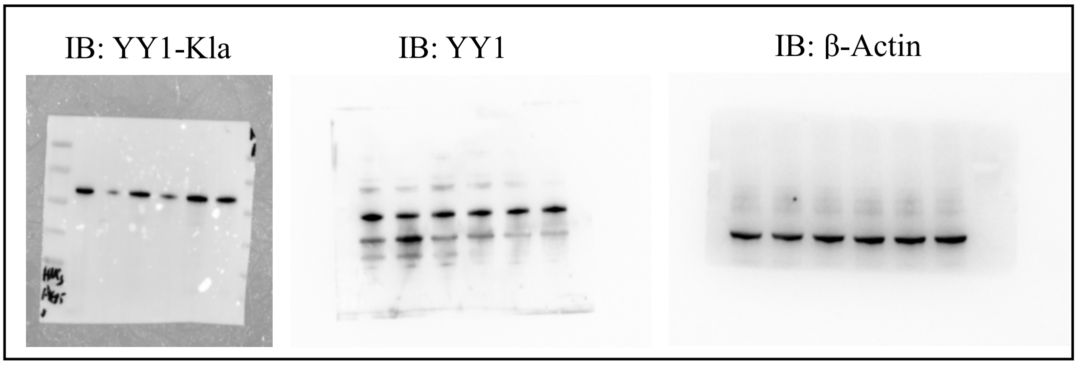

Main Fig. 7a

IB: YY1-Kla

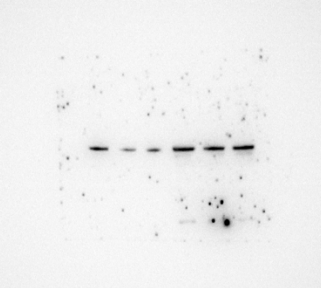

IB: β-Actin

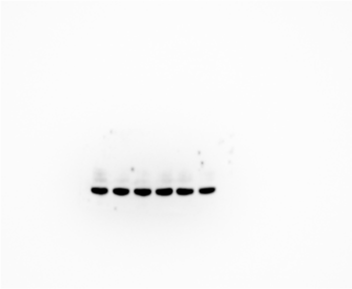

IB: p300

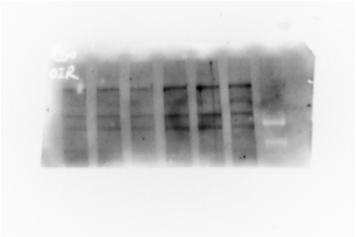

IB: YY1

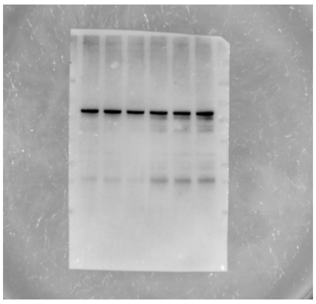

IB: β-Actin

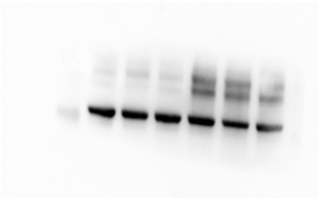

Main Fig. 7c

IB: YY1

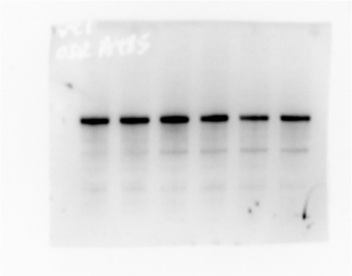

IB: YY1-Kla

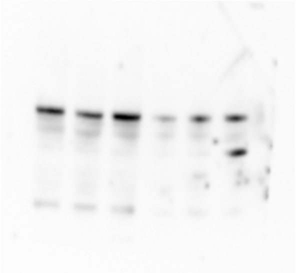

IB: β-Actin

IB: FGF2

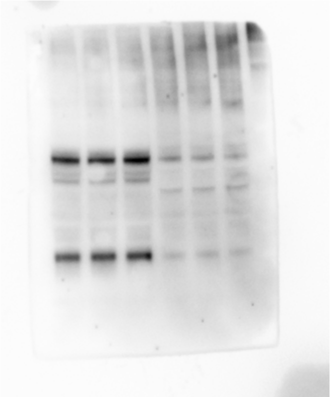

Supplementary Fig. S2f

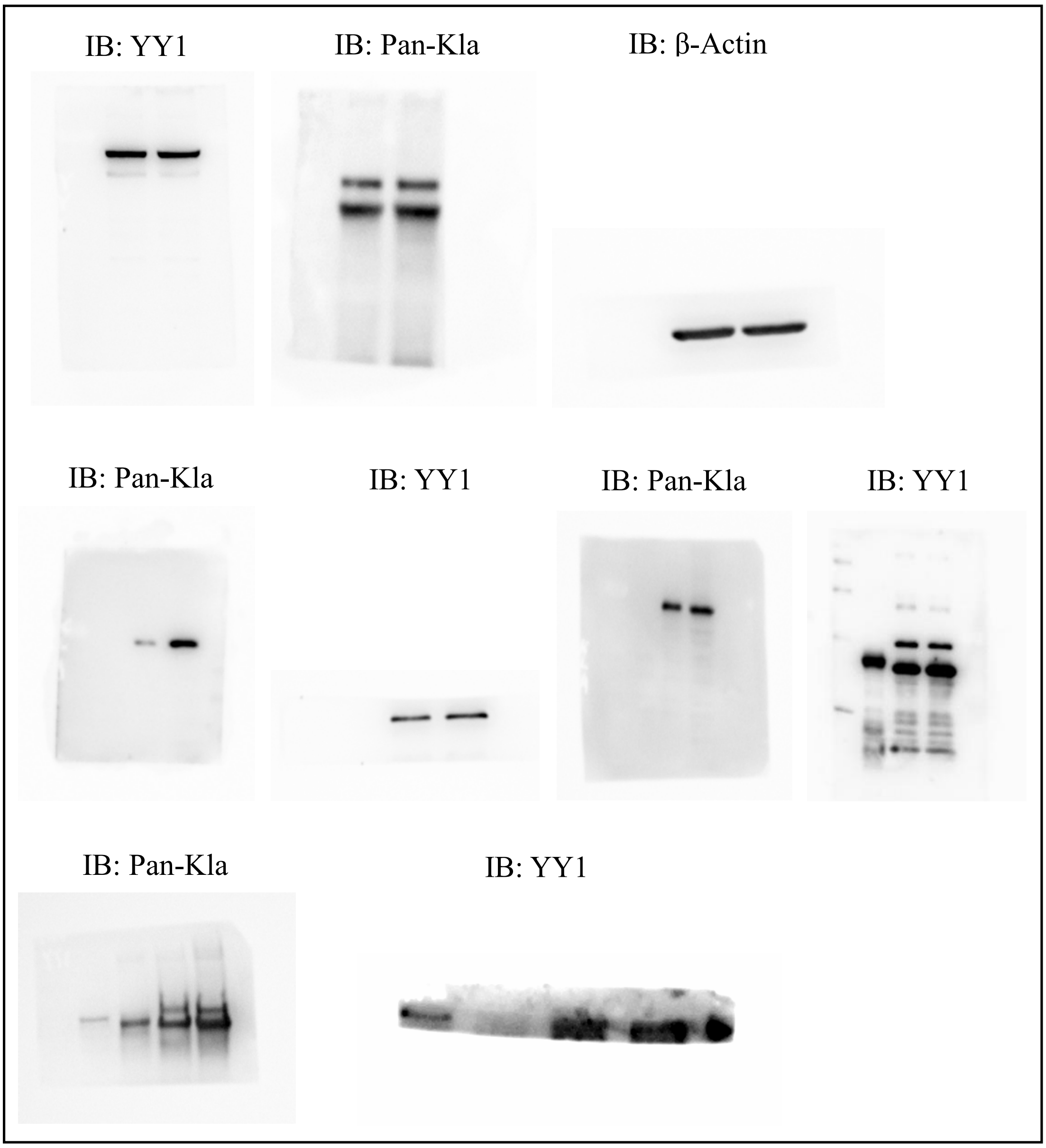

Supplementary Fig. S2h

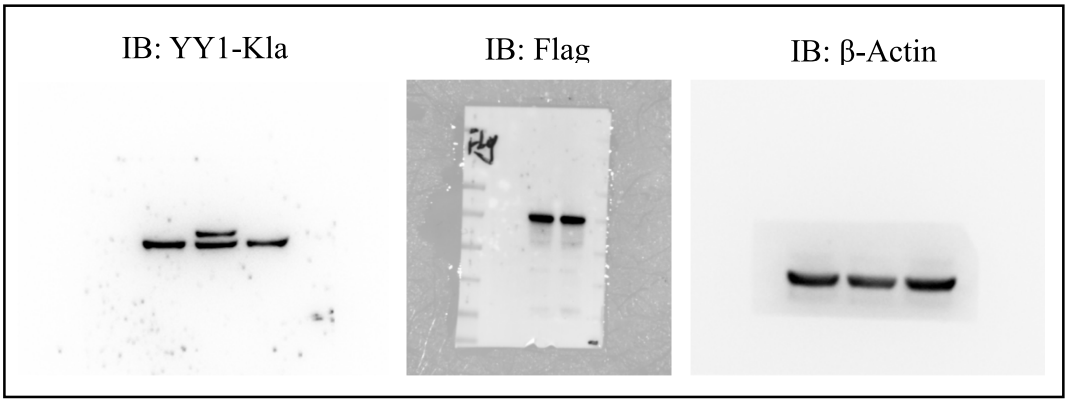

Supplementary Fig. S4a

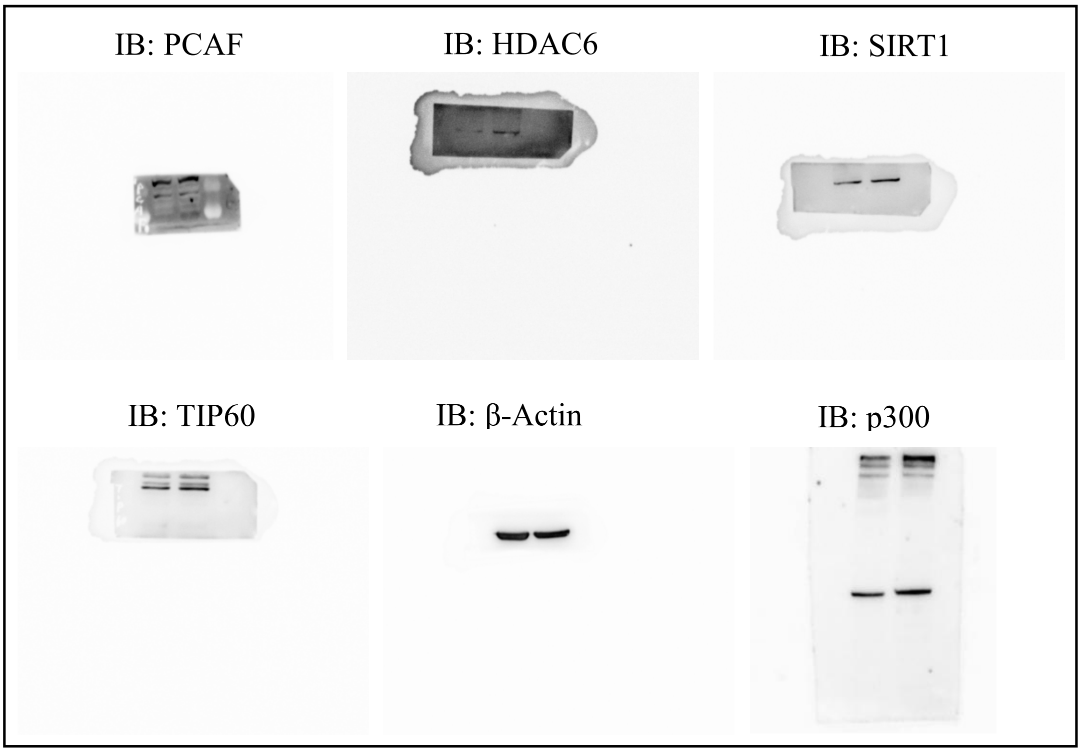

Supplementary Fig. S4b

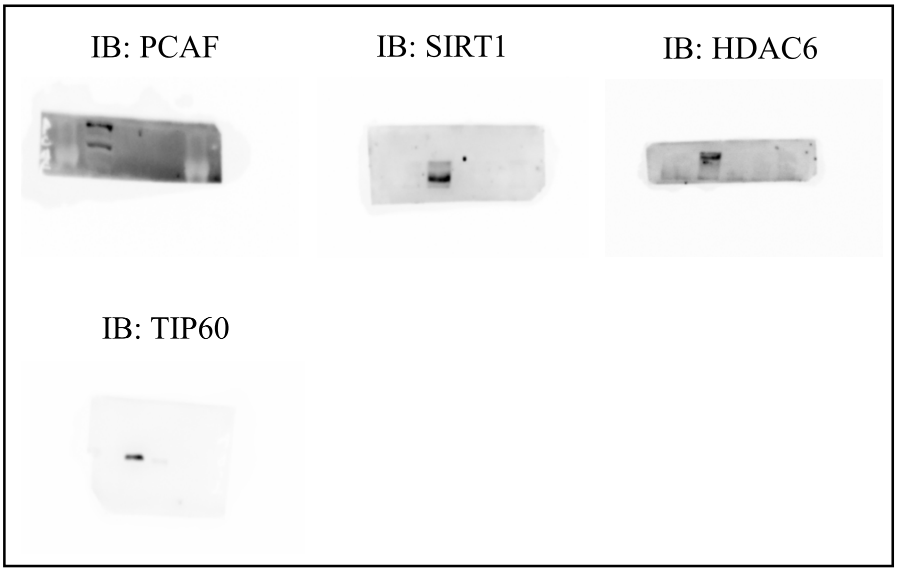

Supplementary Fig. S4c

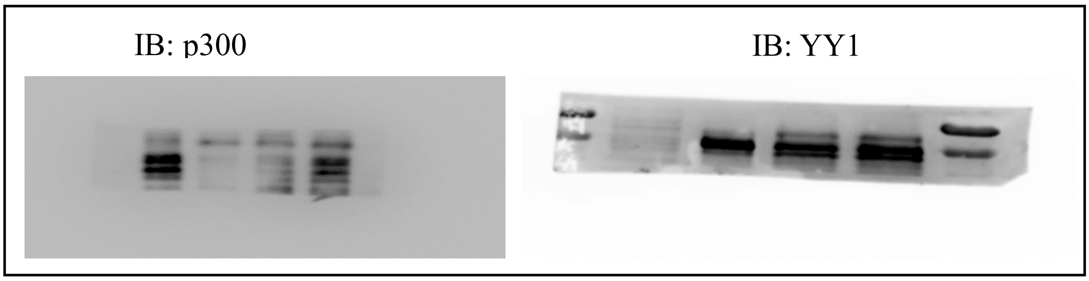

Supplementary Fig. S4d

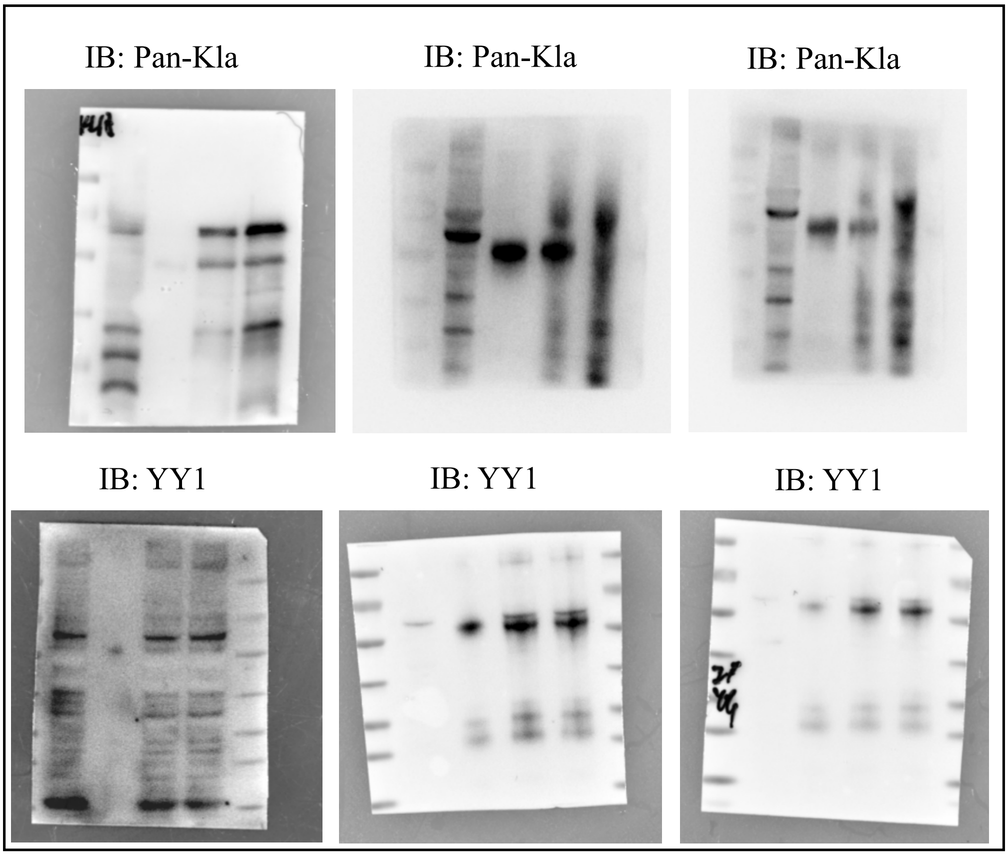

Supplementary Fig. S4e

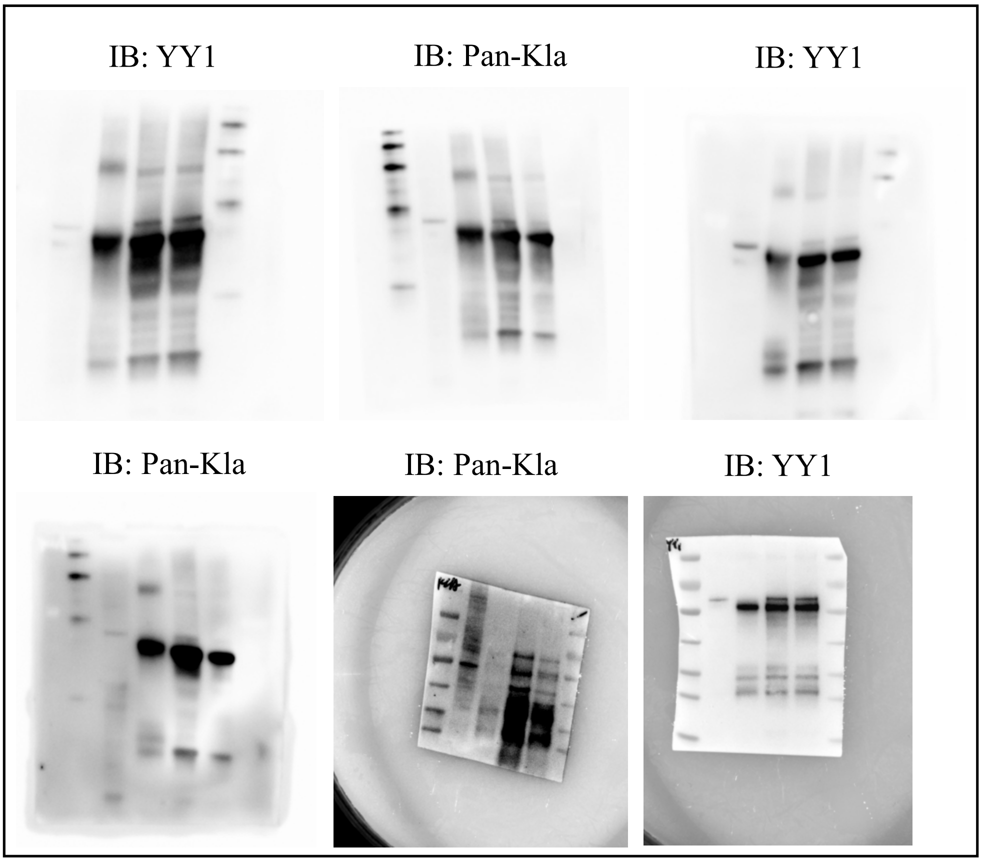

Supplementary Fig. S5a

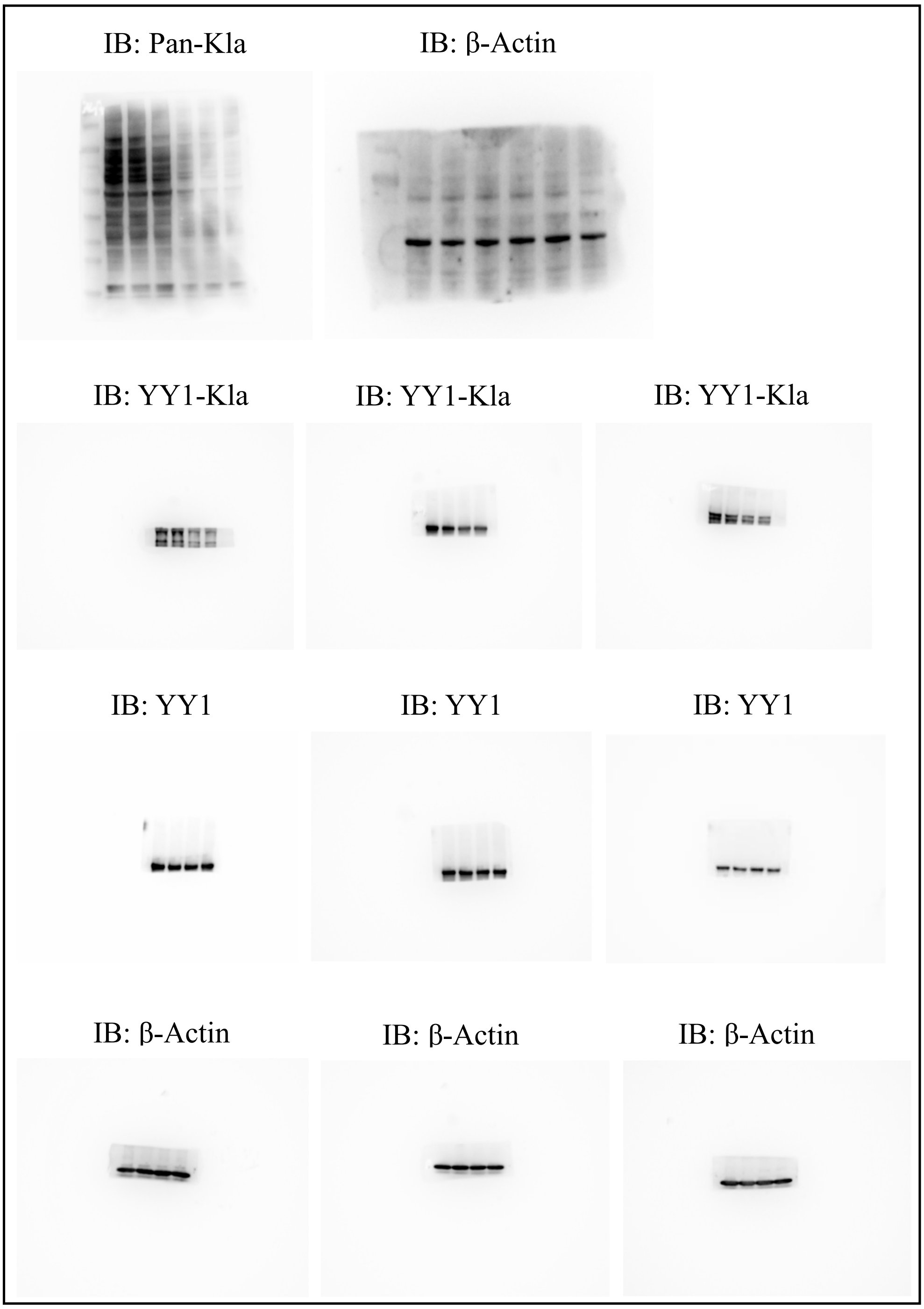

Supplement: Supplementary file 4 — Additional file 4. Uncropped blot images. [file 13059_2023_2931_MOESM4_ESM.pdf]
