# Supplementary material for: YY1 lactylation in microglia promotes angiogenesis through transcription activation-mediated upregulation of FGF2
Source: Genome Biol. 2023 Apr 21;24:87. doi: 10.1186/s13059-023-02931-y (PMC10120156; doi:10.1186/s13059-023-02931-y)
Supplement: Supplementary file 5 — Additional file 5. Review history. [file 13059_2023_2931_MOESM5_ESM.docx]

**1^st^ round**

**Reviewer 1**

In this manuscript, authors identified that YY1 lactylation in microglia was important for hypoxia-induced pathological angiogenesis in vitro and in vivo. Mechanistically, YY1 lactylation facilitated its binding on FGF2 promoter upon the mediation of p300, and subsequent FGF2 expression. Overall, this is an interesting paper and is a good addition to the field of angiogenesis. While the phenotype data appear mostly solid, the mechanism analysis needs some improvements.

1. A major concern is the target of YY1 lactylation in microglia and the angiogenic microenvironment. It is debatable about the role of FGF in angiogenesis. A number of studies reported that FGF deficiency in vivo had little effect on angiogenesis (doi: 10.1073/pnas.95.10.5672, doi: 10.1038/nm0298-201, doi: 10.1128/MCB.20.3.979-989.2000, doi: 10.1128/MCB.20.6.2260-2268.2000) and FGF2 may play a major role in normoxic angiogenesis (doi: 10.1073/pnas.2202631119). So far, VEGF-A was considered as the most important angiogenic growth factor in vivo. Therefore, authors need to double confirm FGF2 is the very target of YY1 and exclude the regulation on the other angiogenic growth factors.
2. The pathological ROP model was employed in the present study, which closely correlates to the pathological retinal angiogenesis in clinic. Authors are suggested to investigate the YY1 lactylation in retina developmental angiogenesis, which may extend the scientific significance of the mechanistic findings in general physiological angiogenesis.
3. In regards of the angiogenic assays, low power images and related statistics in transmigration assay should be employed for an objective evaluation. More importantly, spheroid sprouting assay is better to mimic the endothelial angiogenesis in vivo.
4. Deeper investigation of the role of YY1 delactylation in vivo is necessary. Thus, it is worth to examine the retinal angiogenesis in mice with knock-in or overexpression of WT/delactylated YY1 forms in microglia.
5. Is the interaction between YY1 and p300 responsive to hypoxia?
6. EMSA assays could be a good addition to double confirm the differential DNA bindings.

**Reviewer 2**

Wang and colleagues identified multiple hyperlactated proteins and focused on the roles of YY1 transcription factor in the induction of FGF2 expression and pathological angiogenesis in this manuscript titled "YY1 lactylation in microglia promotes angiogenesis through transcription activation mediated upregulation of FGF2". The lactylation, a new lactate-derived post-translational modification, was discovered to play an important role for the first time in retinal neovascular diseases. This finding is innovative. The identification of additional therapeutic targets for the treatment of proliferative retinal diseases is important and potentially clinically useful. This is a very important and interesting study. The manuscript is well written, and the experimental design is good. Some questions and issues should be addressed by the authors before publication.

1. The authors state that 77 sites of 67 proteins with increased lactylation and 190 sites of 162 proteins with decreased lactylation were identified. How many lactylated peptides and proteins were identified in total? Please provide the quality control data of the peptides.
2. Please provide the fold change of lactylation level of YY1 under normal and hypoxic conditions according to sequencing results.
3. Specialized antibodies that target modified peptides were widely used. The authors used specialized YY1-K183 lactylation antibody to verify the results that the lactylation level of YY1 was upregulated in hypoxia, which is consistent with the sequencing results. Are there quality control data to support that this antibody specially target lactylated YY1? If so, please provide this information.
4. Page. 7, lines 144-147. “DCA can reduce the production of lactate ……glycolysis and increases the content of lactate” References are needed to indicate the functions of DCA and Rotenone.
5. Page. 16, lines 315-317: “Microglia can sense physiological and pathological cues, ......” – references needed here.
6. Please indicate the gender of the mice used for animal experiments.
7. Page. 21, line 427, “IBA1” should be “Iba1^+^”
8. Page. 27, line 551: "matching criteria......" – there should a detailed description of the method of the matching criteria.
9. The authors state that mice were intraperitoneally injected with PLX3397 (1mg/(kg·day)) from P10 to P17. However, the time schedule of administration is missing.
10. Regarding the quantification of lactate levels, how long was the incubation time and temperature for the reducing agent solution?
11. Figure 1b, there should be a scale bar.
12. Figure 2d: From which layer were the images taken? Please indicate this.
13. Please improve the quality of the fluorescence image in Figure 3e.
14. The font size of Figure 4(d) is too small.
15. The language of the manuscript should be improve by a native English speaker. Punctuation should be checked carefully.

**Reviewer 3**

Comments:
Methods: Overall the methods were well described and were suitable to address the aims of the study. However, some of the methods were not clearly described in the text. For example, it was not clear how YY1-Kla was determined, by co-IP or by directly detected Kla-YY1? where was the antibody from? The Mass-Spect in Fig. 4d was not clearly labelled. Which peak represented the lactyl conjugated K in YY1?
Conclusion: The most of data support the conclusion. One concern is that VEGF is known target of YY1. How the author excluded VEGF?
Details: most of details were provided. Some as indicated above were needed to clarify.
Significance: The concept that lactylated YY1 contribute to FGF2 expression and angiogenesis is novel.
Broad interest: The work will have broad interest for researchers in genome science, vascular biology and ophthalmology fields.

**Authors’ response**

Reviewer #1

In this manuscript, authors identified that YY1 lactylation in microglia was important for hypoxia-induced pathological angiogenesis in vitro and in vivo. Mechanistically, YY1 lactylation facilitated its binding on FGF2 promoter upon the mediation of p300, and subsequent FGF2 expression. Overall, this is an interesting paper and is a good addition to the field of angiogenesis. While the phenotype data appear mostly solid, the mechanism analysis needs some improvements.ubcultured into spheres, demonstrating their proliferation and differentiation potential.

1. A major concern is the target of YY1 lactylation in microglia and the angiogenic microenvironment. It is debatable about the role of FGF in angiogenesis. A number of studies reported that FGF deficiency in vivo had little effect on angiogenesis (doi: 10.1073/pnas.95.10.5672, doi: 10.1038/nm0298-201, doi: 10.1128/MCB.20.3.979-989.2000, doi: 10.1128/MCB.20.6.2260-2268.2000) and FGF2 may play a major role in normoxic angiogenesis (doi: 10.1073/pnas.2202631119). So far, VEGF-A was considered as the most important angiogenic growth factor in vivo. Therefore, authors need to double confirm FGF2 is the very target of YY1 and exclude the regulation on the other angiogenic growth factors.

**Re: We appreciate the reviewer’s constructive suggestion. We conducted ChIP-qPCR assays of YY1 under normoxic and hypoxic conditions and confirmed that FGF2 is the very target of YY1 in microglia in response to hypoxia. We found that under hypoxic condition, only the enrichment of YY1 in the promoters of FGF2 was upregulated and didn’t observe in other angiogenic factors in microglia, such as VEGFA, MMP2, MMP9 and ANGPTL6 (Fig. 5g). Meanwhile, K183R mutation of YY1 decreased its transcription of FGF2 and inhibited FGF2 expression (Fig. 5h). These results indicated that hyperlactylation of YY1 induced by hypoxia is responsible for promoting FGF2 expression in microglia. Indeed, multiple angiogenic factors work together to promote neovascularization in the process of pathological retinal angiogenesis. Consistently, in our study, we detected upregulation of both FGF2 and VEGFA in OIR retina, but we only observed downregulation of FGF2 after PLX3397 treatment induced microglia depletion (Fig. 5a, c). Our results and others indicate that various cells contribute to angiogenesis by secreting different angiogenic factors. Müller cells and Endothelial cells are thought to be major cells secreting VEGFA (doi:** **10.1002/path.2611, doi: 10.1073/pnas.92.3.905, doi: 10.1016/j.visres.2017.05.005, doi:10.4239/wjd.v6.i5.726, doi:** **10.1016/0028-3908(76)90091-5, doi: 10.1056/NEJMra1208129 ). Myeloid cell-specific VEGFA knockout didn’t change the VEGFA levels in the retina and had no effect on the progression of oxygen-induced retinopathy (doi:** **10.1016/j.exer.2017.10.011). And we think that FGF2 may be a major factor secreted by retinal microglia and contributes to pathological neovascularization, which was also reported in other’s studies (doi: 10.1073/pnas.2023290118, doi: 10.1159/000453550, doi: 10.1016/s0165-6147(00)01676-x, doi: 1016.2019/j.exer.107775). Meanwhile, we found that PLX3397 treatment resulted in impaired retinal neovascularization, which is reversed by intravitreal injection of recombinant FGF2, further confirming the important role of FGF2 (Fig. S3h). We have added sentences to address this issue (please see page 12, lines 247-254, Fig. 5g and 5h; page 13, lines 256-258, Fig. S3h).**

2. The pathological ROP model was employed in the present study, which closely correlates to the pathological retinal angiogenesis in clinic. Authors are suggested to investigate the YY1 lactylation in retina developmental angiogenesis, which may extend the scientific significance of the mechanistic findings in general physiological angiogenesis.

**Re: We appreciate the reviewer’s constructive suggestion. We explored the level of YY1 lactylation in the retina of WT mouse pups at post-natal early (P5), middle (P10, 15) and late (P20) stages. Mice are born blind and retinal vascular develops after berth. The superficial layer of retinal vessels is generated during the first week after birth while the deep and intermediate vascular networks are formed during the second and third postnatal weeks (10.1167 /iovs.10-5176). During the development of retinal vascular network, the retina is relatively hypoxic, which promotes angiogenesis. Interestingly, we found hyperlactylation of YY1 in the early stage of retinal development . The level of YY1 lactylation decreased at later stage, which indicates that YY1 may also play roles in the physiological angiogenesis (Fig. r1). We have added sentences to address this issue (please see page 15, lines 301-307, Fig. S5b). Further study is necessary to explore it deeply.**

1. In regards of the angiogenic assays, low power images and related statistics in transmigration assay should be employed for an objective evaluation. More importantly, spheroid sprouting assay is better to mimic the endothelial angiogenesis in vivo.

**Re: We appreciate the reviewer’s constructive suggestion. We have provided low power images as suggested, and related statistics have been evaluated objectively by imageJ (Fig. 3e, 3k, 4h, S4h and S4l). And we have performed spheroid sprouting assay as requested in the revised manuscript (please see page 27, lines 549-562; page 8, lines 162-164, Fig. 3d; page 9, lines 170-173, Fig. 3j; page 11, lines 214-216, Fig. 4g; page 14, lines 284-287, Fig S43g and S4k).**

2. Deeper investigation of the role of YY1 delactylation in vivo is necessary. Thus, it is worth to examine the retinal angiogenesis in mice with knock-in or overexpression of WT/delactylated YY1 forms in microglia.

**Re: We appreciate the reviewer’s constructive suggestion. We had tried to overexpress WT/delactylated YY1 forms in retinal microglia in vivo by injecting YY1-overexpressing lentivirus into the retina. But we detected no expression of the marker EGFP and YY1 was failed to be overexpressed, which may be because the low transfection efficiency of lentivirus to primary retinal microglia in situ. Obviously, this is one of the limitations of our study, which is discussed in our manuscript. We hope to take in depth research in the future. We have added sentences to address this issue (please see page 18, lines 370-373).**

3. Is the interaction between YY1 and p300 responsive to hypoxia?

**Re: We appreciate the reviewer’s constructive suggestion. We have performed IB of p300 on IP YY1 in microglia under normoxic and hypoxic conditions. And we found that the interaction of p300 and YY1 was enhanced in response to hypoxia. We have added sentences to address this issue (please see page 13, lines 271-272, Fig. S4c).**

6. EMSA assays could be a good addition to double confirm the differential DNA bindings.

**Re: We appreciate the reviewer’s constructive suggestion. We have performed Chip-qPCR assays to confirm the differential DNA bindings of YY1. Consistent with our previous results, we double confirmed that YY1 directly targets the FGF2 promoter region to induce the transcription of FGF2 and it’s regulated by lactylation at lysine 183 (please see page 12, lines 247-254, Fig. 5g and 5h; page 26, lines 531-532). Future research may be needed to double confirm the differential DNA bindings using EMSA assays.**

**Reviewer #2:**

**General comments:**

Wang and colleagues identified multiple hyperlactated proteins and focused on the roles of YY1 transcription factor in the induction of FGF2 expression and pathological angiogenesis in this manuscript titled "YY1 lactylation in microglia promotes angiogenesis through transcription activation mediated upregulation of FGF2". The lactylation, a new lactate-derived post-translational modification, was discovered to play an important role for the first time in retinal neovascular diseases. This finding is innovative. The identification of additional therapeutic targets for the treatment of proliferative retinal diseases is important and potentially clinically useful. This is a very important and interesting study. The manuscript is well written, and the experimental design is good. Some questions and issues should be addressed by the authors before publication.

1. The authors state that 77 sites of 67 proteins with increased lactylation and 190 sites of 162 proteins with decreased lactylation were identified. How many lactylated peptides and proteins were identified in total? Please provide the quality control data of the peptides.

**Re: We appreciate the reviewer’s constructive comments. For the details of the lactylome data, a total of 6021 peptides and 3071 lactylated peptides were identified. In total, 1601 lactylated sites in 751 proteins were identified in both groups of HMC3 cells, with a localization probability >0.75 and a 1% FDR. Quality control revealed that the distribution of peptides is in a reasonable range. (Please see page 9, lines 181-184, Fig. S2a and S2b ).**

2. Please provide the fold change of lactylation level of YY1 under normal and hypoxic conditions according to sequencing results.

**Re: We appreciate the reviewer’s constructive suggestions. We have provided the fold change of lactylation level of YY1 under normal and hypoxic conditions. (Please see page 10, line 196).**

3. Specialized antibodies that target modified peptides were widely used. The authors used specialized YY1-K183 lactylation antibody to verify the results that the lactylation level of YY1 was upregulated in hypoxia, which is consistent with the sequencing results. Are there quality control data to support that this antibody specially target lactylated YY1? If so, please provide this information.

**Re: We appreciate the reviewer’s constructive comments. We have provided the quality control data of the specialized YY1 Kla antibody. Three lactylated peptides of YY1 (KKSGK-（lactyl）K-SYLSGG; GKKSGK-（lactyl）K-SYLSGG and GGKKSGK-（lactyl）K-SYLSGGAC) were designed and used to immunizing rabbits. Then the antibodies were extracted. Dot blots assays revealed that the purified antibody specially targeted the lactylated peptide of YY1 and didn’t target the unlactyalted YY1 peptide (GGKKSGKKSYLSGGAC), the results** **have been shown as followed Fig. r2. The designed YY1 peptides were verified by mass spectrometry, and these results** **have been shown as followed Fig. r3.**

1. Page. 7, lines 144-147. “DCA can reduce the production of lactate ……glycolysis and increases the content of lactate” References are needed to indicate the functions of DCA and Rotenone.

**Re: We appreciate the reviewer’s constructive comments. We have provided the reference in the revised manuscript. (Please see page 7, lines 143-146).**

2. Page. 16, lines 315-317: “Microglia can sense physiological and pathological cues, ......” – references needed here.

**Re: We appreciate the reviewer’s constructive comments. We have provided the reference in the revised manuscript. (Please see page 16, lines 333-335).**

3. Please indicate the gender of the mice used for animal experiments.

**Re: We appreciate the reviewer’s constructive comments. We have provided the information of the gender of the mice used for animal experiments in the revised manuscript. (Please see page 19, lines 386-388).**

4. Page. 21, line 427, “IBA1” should be “Iba1^+^”

**Re：We appreciate the reviewer’s constructive comments. We have corrected it. (Please see page 22, line 445).**

5. Page. 27, line 551: "matching criteria......" – there should a detailed description of the method of the matching criteria.

**Re: We appreciate the reviewer’s constructive comments. We have provided the detailed description of the method of the matching criteria. (Please see page 28, lines 581-583).**

6. The authors state that mice were intraperitoneally injected with PLX3397 (1mg/(kg·day)) from P10 to P17. However, the time schedule of administration is missing.

**Re: We appreciate the reviewer’s constructive comments. We have provided the time schedule of administration in the method of microglia depletion. (Please see page 20, lines 404-405).**

7. Regarding the quantification of lactate levels, how long was the incubation time and temperature for the reducing agent solution?

**Re: We appreciate the reviewer’s constructive comments. We have provided this information in the method of quantification of lactate levels. (Please see page 21, lines 434-436).**

8. Figure 1b, there should be a scale bar.

**Re: We appreciate the reviewer’s constructive comments. We have added the scale bar. (Please see Fig. 1b).**

9. Figure 2d: From which layer were the images taken? Please indicate this.

**Re: We appreciate the reviewer’s comments. Due to the multiple layers of retinal vascular and the non-uniform thickness of retina, we took the picture of the whole retinal vascular layer superimposed, which was more accurate to present the results of retinal angiogenesis.**

10. Please improve the quality of the fluorescence image in Figure 3e.

**Re: We appreciate the reviewer’s constructive comments. We have updated the quality of fluorescence image in Fig. 3e with better quality. (Please see Fig. 3f).**

11. The font size of Figure 4(d) is too small.

**Re: We appreciate the reviewer’s constructive comments. We have updated the font size in Figure 4d. (Please see Fig, 4d).**

12. The language of the manuscript should be improved by a native English speaker. Punctuation should be checked carefully.

**Re: Re: We appreciate the reviewer’s constructive comments. We thank the reviewer’s suggestions and revise the manuscript carefully. And this manuscript was edited for proper English language by native English-speaking editors at AJE. We have revised the manuscript carefully.**

**“of” to “in” (Please see page 2, line 34)**

**“at exploring” to “to explore” (Please see page 2, line 36)**

**“finding” to “identify” (Please see page 2, line 36)**

**“the” to “an” (Please see page 2, line 38)**

**“Co-cultures” to “Cocultures” (Please see page 2, line 39)**

**“to” to “on” (Please see page 2, line 40)**

**“Immunofluorence” to “Immunofluorescence” (Please see page 2, line 42)**

**“Microglia” to “Microglial” (Please see page 2, line 45)**

**“promoted” to “promoting” (Please see page 2, line 47)**

**“Post-translational modifications (PTM)” to “Posttranslational modifications (PTMs)” (Please see page 3, line 61).**

**“clinic” to “clinical practice” (Please see page 4, line 76)**

**“And the repeated intra-ocular” to “Repeated intraocular” (Please see page 4, line 76)**

**“achieves its effect by destructive effects and” to “is a destructive method that” (Please see page 4, line 78)**

**“mechanistical” to “mechanistic” (Please see page 5, line 104).**

**“level” to “levels” (Please see page 7, line 147)**

**“worthywhile question” to “worthy of investigation” (Please see page 18, line 373)**

**“transcription” to “transcriptional” (Please see page 19, line 379).**

**Reviewer #3:**

**Comments:**

1. Methods: Overall the methods were well described and were suitable to address the aims of the study. However, some of the methods were not clearly described in the text. For example, it was not clear how YY1-Kla was determined, by co-IP or by directly detected Kla-YY1? where was the antibody from? The Mass-Spect in Fig. 4d was not clearly labelled. Which peak represented the lactyl conjugated K in YY1?

**Re: We appreciate the reviewer’s constructive comments. We have used both IB after IP assays (IP YY1, IB Pan-Kla) and specific YY1 lactylation antibody (YY1-Kla) to verify the level of YY1-lactylation.The specific YY1 lactylation antibody was customized by PTM BIOLABS and dot blots was used to confirm the specificity of this antibody (Fig. 2 and 3，please see Reviewer 2, Q3). The results obtained by these two methods were consistent. The mass shift of 72.021 Daltons on lysine residues was thought to be lactylated site (doi: 10.1038/s41586-019-1678-1). For example, in Fig 4d, the mass spectrometry molecular weight of b4 (K+S+Y+L) was 564.3, which is 72.74 Daltons higher than the theory weight (491.56). We have marked the represented lactyl conjugated K in YY1. We also added sentences to address this issue (please see page 22, lines 448-449; page 10, lines 206-208, Fig. 4e, 4d and S2f).**

2. The most of data support the conclusion. One concern is that VEGF is known target of YY1. How the author excluded VEGF?

**Re: We appreciate the reviewer’s constructive comments. For this question, please see Reviewer 1, Q1. Indeed, it’s evident that various angiogenic factors work together to promote neovascularization in the process of pathological retinal angiogenesis. In our study, we also detected upregulation of both FGF2 and VEGFA in OIR retina, but we only observed downregulation of FGF2 in PLX3397 treated mice. Previous studies indicated that various cells contribute to angiogenesis by secreting different angiogenic factors. VEGFA was mainly secreted by Müller cells and Endothelial cells (doi:** **10.1002/path.2611, doi: 10.1073/pnas.92.3.905, doi: 10.1016/j.visres.2017.05.005, doi:10.4239/wjd.v6.i5.726, doi:** **10.1016/0028-3908(76)90091-5, doi: 10.1056/NEJMra1208129 ). Myeloid cell-specific VEGFA knockout had no effect on the expression of VEGFA in the retina and didn’t affect the progression of oxygen-induced retinopathy (doi:** **10.1016/j.exer.2017.10.011). We think FGF2 may be a major factor secreted by retinal microglia and contributes to pathological neovascularization, which was also reported by other’s studies.  (doi: 10.1073/pnas.2023290118, doi: 10.1159/000453550, doi: 10.1016/s0165-6147(00)01676-x). To further confirm that FGF2 is target of YY1, we conducted CHIP-qPCR assays of YY1 under normoxic and hypoxic conditions and found that only the enrichment of YY1 in the promoters of FGF2 was upregulated and didn’t observe in other angiogenic factors in microglia. Meanwhile, K183R mutation of YY1 decreased its transcription of FGF2 and inhibited FGF2 expression. These results indicated that hyperlactylation of YY1 induced by hypoxia is responsible for promoting FGF2 expression in microglia.**

We thank you and the reviewers’ time spent on this manuscript. These comments have definitely improved the manuscript, and we hope it is now in a form acceptable for publication in ***Genome Biology***.

**2nd round**

**Reviewer 1**

The authors have addressed most of my concerns properly. However, they did not provide any direct evidences for demonstrating the role of YY1 delactylation in vivo, which should be an important piece to support the conclusion of the study. They may check the serum type of the lentivirus employed or choose AAV, genetic strategy and so on.

**Reviewer 2**

The authors have addressed all my concerns and I have no further questions. I recommend the publication of this important article.

**Reviewer 3**

The authors have addressed my previous concerns.
